# Sub national variation and inequalities in under-five mortality in Kenya since 1965

**DOI:** 10.1186/s12889-019-6474-1

**Published:** 2019-02-04

**Authors:** Peter M. Macharia, Emanuele Giorgi, Pamela N. Thuranira, Noel K. Joseph, Benn Sartorius, Robert W. Snow, Emelda A. Okiro

**Affiliations:** 1 Population Health Unit, Kenya Medical Research Institute-Wellcome Trust Research Programme, Nairobi, Kenya; 2 Lancaster Medical School, Lancaster University, Lancaster, UK; 3 Public Health Medicine, School of Nursing and Public Health, University of KwaZulu-Natal, Durban, South Africa; 4 Centre for Tropical Medicine and Global Health, Nuffield Department of Clinical Medicine, University of Oxford, Oxford, UK

**Keywords:** Under-five mortality, Sub national, Variation, Inequalities, Spatio-temporal, Kenya

## Abstract

**Background:**

Despite significant declines in under five mortality (U5M) over the last 3 decades, Kenya did not achieve Millennium Development Goal 4 (MDG 4) by 2015. To better understand trends and inequalities in child mortality, analysis of U5M variation at subnational decision making units is required. Here the comprehensive compilation and analysis of birth history data was used to understand spatio-temporal variation, inequalities and progress towards achieving the reductions targets of U5M between 1965 and 2013 and projected to 2015 at decentralized health planning units (counties) in Kenya.

**Methods:**

Ten household surveys and three censuses with data on birth histories undertaken between 1989 and 2014 were assembled. The birth histories were allocated to the respective counties and demographic methods applied to estimate U5M per county by survey. To generate a single U5M estimate for year and county, a Bayesian spatio-temporal Gaussian process regression was fitted accounting for variation in sample size, surveys and demographic methods. Inequalities and the progress in meeting the goals set to reduce U5M were evaluated subnationally.

**Results:**

Nationally, U5M reduced by 61·6%, from 141·7 (121·6–164·0) in 1965 to 54·5 (44·6–65·5) in 2013. The declining U5M was uneven ranging between 19 and 80% across the counties with some years when rates increased. By 2000, 25 counties had achieved the World Summit for Children goals. However, as of 2015, no county had achieved MDG 4. There was a striking decline in the levels of inequality between counties over time, however, disparities persist. By 2013 there persists a 3·8 times difference between predicted U5M rates when comparing counties with the highest U5M rates against those with the lowest U5M rates**.**

**Conclusion:**

Kenya has made huge progress in child survival since independence. However, U5M remains high and heterogeneous with substantial differences between counties. Better use of the current resources through focused allocation is required to achieve further reductions, reduce inequalities and increase the likelihood of achieving Sustainable Development Goal 3·2 on U5M by 2030.

**Electronic supplementary material:**

The online version of this article (10.1186/s12889-019-6474-1) contains supplementary material, which is available to authorized users.

## Background

Under-five mortality (U5M), the probability that a child will die before reaching the age of five, is a benchmark of a country’s health status and progress towards achievement of development goals [1, 2]. Tracking U5M as part of international development targets began with the World Summit for Children in 1990, which set goals to reduce U5M by a third and/or to 70 per 1000 live births between 1990 and 2000 [3]. This was followed by the Millennium Development Goal (MDG) 4 that proposed a reduction of U5M by two-thirds between 1990 and 2015 [4]. Currently, as part of the Sustainable Development Goals (SDGs), goal 3·2 seeks to end preventable deaths of children under-5 years of age and to reduce U5M to 25 per 1000 live births by 2030 [5].

It has been estimated that there has been a 53% reduction in U5M globally between 1990 and 2015 [1, 6]. The slowest reductions in U5M were observed in sub-Saharan Africa (SSA). Only 11 countries out of 48 in SSA achieved the MDG 4 target by 2015 [6]. International benchmarking of national U5M developments goals has improved with time, with increasing data and improved methodologies. In recent years, new methodologies have been applied to understand within-country temporal and spatial heterogeneity in child survival [7]. However, examples of how U5M has changed at sub-national levels remains limited to only a few SSA countries and imperfectly described [8–12]. In all countries, disease burdens are expected to be concentrated in a few sub-national areas; identification of these areas and directing suitable interventions to these areas will accelerate national child mortality reductions and ensure effective and equitable resource allocation.

Sub-national variations of child survival in Kenya were first described during the 1980s [13–16]. Advances in spatio-temporal statistics and analysis of multiple demographic datasets have provided new opportunities to study spatial and temporal heterogeneities of child survival across Kenya [1, 7, 17, 18]. However, recent analyses have not harnessed all the available sample surveys and census data, nor have they always provided annual predictions at sub-national units required for decentralized policy making since Kenya’s independence. In addition, assessments of progress towards achieving global milestones and reducing inequalities in U5M at subnational units has not been attempted in Kenya.

Here the analysis of all available birth history data from household surveys and population census data is presented to provide annual predicted quantities of U5M since 1965 at each of Kenya’s counties created in 2010 for decentralized health agenda. The estimated rates were used to express spatial and temporal heterogeneities in demographic transitions toward sub-national U5M goals and the long-term inequalities in child survival.

## Methods

### Country context

At the turn of the last century, the U5M rate was approximately 250 deaths per 1000 live births in Kenya [14]. Following the establishment of a rudimentary health system, U5M had declined to about 239 per 1000 live births by 1962 [13, 14]. After independence in 1963, the government adopted socio-economic policies to alleviate hunger, ignorance and disease via the creation of equal opportunities for all citizens and an equitable distribution of wealth opportunities [19].

Twenty years after independence, Kenya began to launch policies to specifically improve child survival, notably in line with the Alma-Ata declaration in 1978 on primary health care. The Kenya expanded programme on immunization (EPI) was established in 1980 to coordinate immunization services for six childhood killer diseases, at the time including tuberculosis, polio, diphtheria, whooping cough, tetanus and measles [20]. However, during the late 1980s through to late 1990s, Kenya was affected by increasing prevalence of HIV [21], reintroductions of user fees in 1992 [22], poor coverage of basic healthcare services [23] and re-emerging malaria epidemics as a result of widespread resistance to Chloroquine [24].

At the beginning of 2000, there were numerous reproductive, maternal, newborn and child health (RMNCH) policies aligning the health agenda with MDGs [23, 25]. Additional vaccines including Yellow fever, Hepatitis B and Haemophilus Influenza B type were added to the EPI bundle and user fees abolished in peripheral health facilities in 2004. Intensified efforts against malaria were mobilized under the Roll Back Malaria (RBM) initiative including replacing failing drugs with efficacious artemisinin-based combination (ACT) drugs and expanded, free delivery of insecticide-treated bed nets (ITNs) from 2006 [26, 27]. Free preventative interventions for HIV including preventing mother-to-child transmission and antiretroviral therapy were also expanded significantly from 2006 [21]. Pneumococcal conjugate vaccine was introduced in 2011 while rotavirus and measles second dose vaccines were introduced in 2014 and 2013 respectively. Since 2013, all services at government outpatient facilities and maternity services have been offered free of charge [22]. In addition, there has been renewed interests in expanding primary healthcare services as close to homesteads as possible through the Malezi Bora (good upbringing) initiative [28], the Beyond Zero [29] initiative and the universal health systems initiative focusing on RMNCH services among the underserved population [30].

**Fig. 1:**
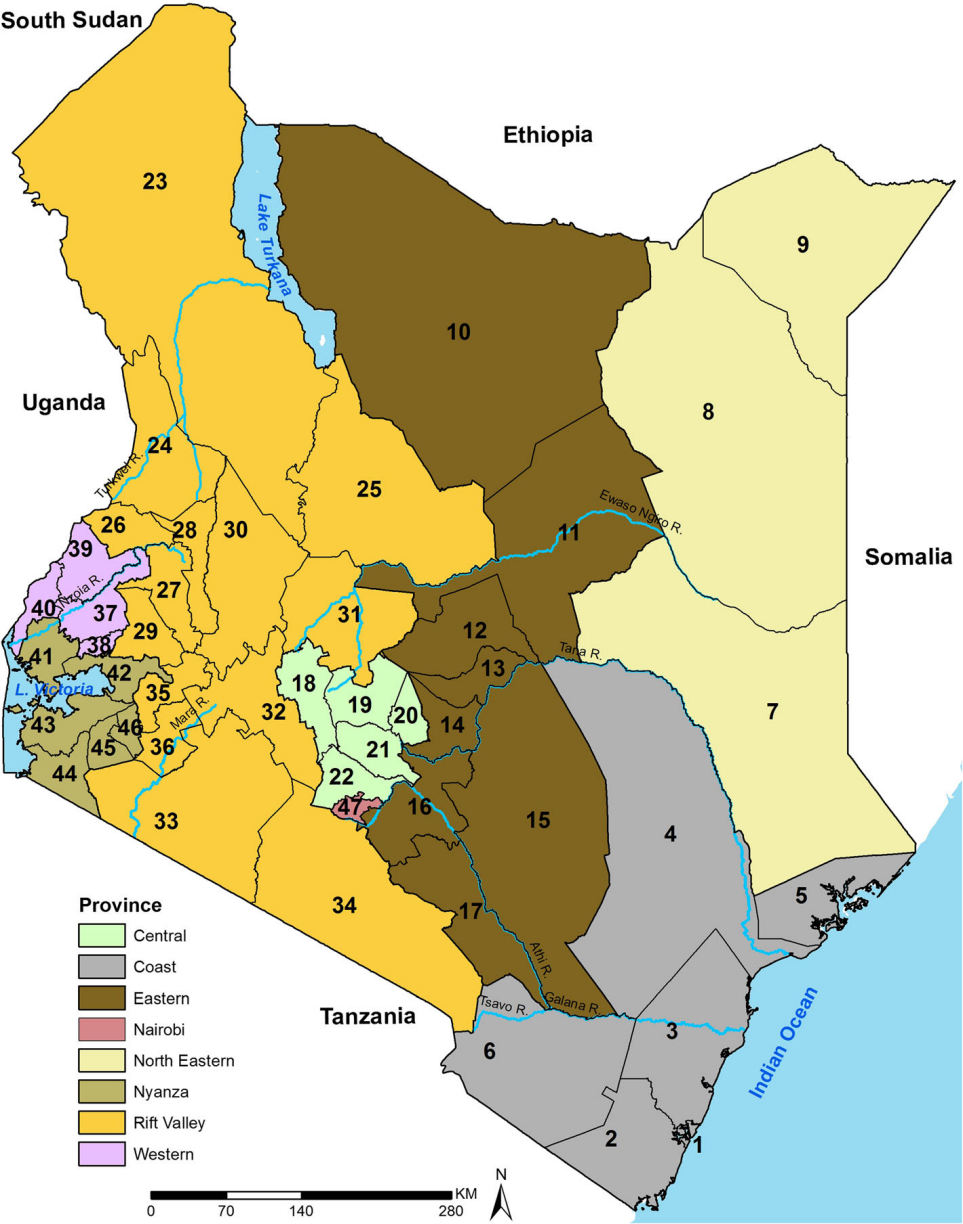
Map of Kenya showing 8 provinces (colored) and the 47 sub-national units (counties) as dark lines water bodies and major rivers are shown in blue**.** Coast province: Mombasa [1], Kwale [2], Kilifi [3], Tana River [4], Lamu [5], Taita Taveta [6]; North Eastern province: Garissa [7], Wajir [8], Mandera [9]; Eastern province: Marsabit [10], Isiolo [11], Meru [12], Tharaka Nithi [13], Embu [14], Kitui [15], Machakos [16], Makueni [17]; Central province: Nyandarua [18], Nyeri [19], Kirinyaga [20], Murang’a [21], Kiambu [22]; Rift Valley province: Turkana [23], West Pokot [24], Samburu [25], Trans Nzoia [26], Uasin Gishu [27], Elgeyo Marakwet [28], Nandi [29], Baringo [30], Laikipia [31], Nakuru [32], Narok [33], Kajiado [34], Kericho [35], Bomet [36]; Western province: Kakamega [37], Vihiga [38], Bungoma [39], Busia [40]; Nyanza province: Siaya [41], Kisumu [42], Homa Bay [43], Migori [44], Kisii [45], Nyamira [46]; Nairobi province: Nairobi [47]

2010 was a landmark year for Kenya when a new constitution was adopted which revised units of administration and health planning to 47 county governments (Fig. 1), including county ministries of health with broad policy directions maintained at the national level. The decentralized system came into effect in 2013 after the general elections and now represent units for federal policy planning to support the SDGs [31]. The counties cover variable climatic, ecological, socio-economic and infrastructure conditions. These dissimilarities govern the rainfall patterns, agricultural activities and human settlement with the highest population densities in the counties around Lake Victoria, central and the coastal areas while the southern and northern areas of Kenya are sparsely populated.

### Data assembly

The ambition of U5M data assembly was to resolve all available data at sub-national resolutions (county) over time. Sample household survey and population census data with either summary birth history (SBH) and/or complete birth history (CBH) collected between 1989 and 2014 in Kenya were assembled (Additional file 1). All the datasets were reconciled to the 47 counties boundaries (Fig. 1) as they were spatially misaligned over time (Additional file 2). The birth histories, maternal age, and/or time since first birth were collected through questions posed to women aged between 15 and 49 years. CBH records the date of each live birth and the date of death for those who had not survived while SBH records aggregated information on children ever born alive and who had since died [32]. Thirteen (13) national and/or sub-national datasets were available from the Kenya National Bureau of Statistics (KNBS) data archive [33], Integrated Public Use Microdata Series (IPUMS) [34], Multiple Indicator Cluster Survey (MICS) [35] and the Demographic and Health Survey (DHS) [36] data portal (Table 1).

**Table 1:** Surveys and censuses undertaken in Kenya with data on either summary birth history (SBH) and/or complete birth history (CBH) since 1989 comprising of six Demographic and Health Surveys (DHS), four Multiple Indicator Cluster Surveys (MICS) and three population censuses. Table includes the number of counties covered, the sample size by source, and the exposure variable: whether maternal age (MA), and/or time since first birth (TFB) variables were collected

Survey	Year of Survey	Counties with data	Female (15–49 yrs)	Birth History	Exposure
Demography and Health Survey (DHS)	1989	38	7150	SBH and CBH	MA and TFB
	1993	40	7540	SBH and CBH	MA and TFB
	1998	38	7881	SBH and CBH	MA and TFB
	2003	47	8195	SBH and CBH	MA and TFB
	2008/09	47	8444	SBH and CBH	MA and TFB
	2014	47	31,079	SBH and CBH	MA and TFB
Multiple Indicator Cluster Survey (MICS)	2000	45	10,537	SBH	MA and TFB
	2007	3	881	SBH	MA and TFB
	2008	8	13,606	SBH	MA only
	2011	6	5908	SBH and CBH	MA and TFB
Population census	1989–5%	47	238,027	SBH	MA only
	1999–5%	47	345,647	SBH	MA only
	2009–10%	47	934,904	SBH	MA only

2009 MICS conducted in the informal settlements of Mombasa was excluded since U5M in urban areas was not represented while 2013/14 MICS covering three counties was excluded due to under reporting of under-5 deaths

### Under five mortality computation

The household surveys and censuses data included various levels of information (Table 1). One direct and four independent indirect demographic methods were used to analyze CBH and SBH data respectively (Additional file 3). Using the direct method, the months a child lived before death or age five were allocated to six age-groups [0–1, 1–11, 12–23, 24–35, 36–47, 48–59 months] and two-year time-periods. The monthly probability of survival *(P)* was calculated as the ratio of deaths to the number of months lived in each group and period raised to a power equal to the number of months in the age-group. U5M for each period (_*q*_5_*t*_) was computed by subtracting from one the product of survival probabilities per period [32, 37] (Eq. 1) (Additional file 3). National trends were analyzed using the direct method since CBH does not require a life table model and the surveys are representative nationally.

1 $$ q{5}_t=1-\left\{{P_{t, mo.0}}^{\ast }{\left({P}_{t, mo s.1-11}\right)}^{11\ast }{\left({P}_{t,\mathrm{yr}\ 1}\right)}^{12\ast }{\left({P}_{t,\mathrm{yr}\ 2}\right)}^{12\ast }{\left({P}_{t,\mathrm{yr}\ 3}\right)}^{12\ast }{\left({P}_{t,\mathrm{yr}\ 4}\right)}^{12}\right\} $$

The four indirect techniques included, two cohort and two period methods, indexed by either maternal age or time since first birth [32] adapted from Brass/Trussell models converting the proportion of children dead (CD) to an U5M rate [38]. Using the cohort approach, women were grouped in 5-year cohorts of age or time since first birth, the CD was computed per cohort and converted to U5M rate using eq. 2. The regression coefficients (*β*_1*i*_ − *β*_4*i*_) and the country random effect (Uij) were generated from CBH obtained from household surveys across SSA [32] (Additional file 3).

2 $$ \mathrm{logit}\left({{}_5q}_{ij k}\right)={\beta}_{0i}+{U}_{ij}+{\beta}_{1i}\mathrm{logit}\left(\frac{CD_{ij k}}{CEB_{ij k}}\right)+{\beta}_{2i}{CEB}_{ij k}+{\beta}_{3i}\frac{P{\left(15-19\right)}_{jk}}{P{\left(20-24\right)}_{jk}}+{\beta}_{4i}\frac{P{\left(20-24\right)}_{jk}}{P{\left(25-29\right)}_{jk}}+{\varepsilon}_{ij k} $$

Where (j) indexes a country, (k) a survey, (i) a cohort, CD is children dead, CEB is Children Ever Born while (P) are parity ratios.

Limitations in the cohort approach were addressed through period techniques that account for U5M experiences of both young and older women when calculating U5M estimates for the most recent time and generating predictions for up to 25 years prior to a survey unlike cohort’s 18 years [32]. Period methods generates children dead and children ever born for each year prior to the survey and converts it to a population level U5M (Eq. 3). The maternal age and time since first birth regional empirical distributions of births and deaths were used to redistribute reported births and deaths from each survey and census. The CD was computed and converted to an U5M rate for years prior to the survey using regional empirical coefficients (*β*^0^_*t*_, *U*_*tj*_and *β*^1^_*t*_) via eq. 3 [32] (Additional file 3).

3 $$ Logit\left(5q{0}_{ijk}\right)={\beta^0}_t+{U}_{tj}+{\beta^1}_t\log it\left(\frac{CD_{tj k}}{CEB_{tj k}}\right)+{\varepsilon}_{ijk} $$

Estimations were done in StataCorp. 2014 [Stata Statistical Software: Release 14. College Station, TX: StataCorp LP] by survey and county without pooling data to avoid over-smoothing of trends and allow detection of data quality issues on a case by case basis. Cohort methods allowed for the computation of U5M rates up to 18 years prior to a survey while period methods allowed for prediction of up to 25 years before the survey [32]. Due to the diversity of methods and data sources used, for each county for any single time point, there were multiple estimates of U5M requiring spatio-temporal modelling to smooth trends.

### Bayesian spatio-temporal modelling

In order to borrow the strength of information in U5M estimates across time and space, while accounting for heterogeneity between surveys and the different techniques used for U5M estimation, a Bayesian spatio-temporal Gaussian process regression model [39, 40] with a heteroscedastic error component was developed (Eq. 4). Specifically, the latter was modelled as a Gaussian noise (*Z*_*ijkt*_) whose variance was the product of the log-transformed sample size from a given survey, county and year, and a second factor that accounts for the variability inherent to the demographic methods used to quantify U5M. The log of the sample size was used in order to obtain equally distributed weights to borrow information across all surveys and avoid excessive overweighting of the census data. The spatio-temporal correlation was specified using a separable structure, given by the product of a conditionally autoregressive structure in space and an autoregressive process of the first order in time (Additional file 3).

4 $$ \log \left\{\frac{Q_{ijkt}}{1-{Q}_{ijkt}}\right\}=\alpha +{S}_{kt}+{Z}_{ijkt} $$

A Gaussian distribution was used for the logit-transformed U5M (*Q*_*ijkt*_) from the *i*^*th*^ survey (*n* = 13), based on demographic method *j* (*n* = 5) for the *k*^*th*^ county (*n* = 47) and year *t* (*n* = 49), 1965–2013. *S*_*kt*_ is a spatio-temporal Gaussian process and *Z*_*ijkt*_ is Gaussian noise.

The model was fitted using a bespoke Markov Chain Monte Carlo algorithm, developed in the R software environment (Version 3·4·1), that generated 10,000 predictive samples of smoothed U5M rates by county and year. To reduce the between-samples correlation, the algorithm was iterated for 110,000 times and retained every 10th sample after a burn-in of 10,000 samples. To assess the predictive performance of the model, a cross-validation procedure was used with a randomly selected hold-out sample containing 10% of the observed U5M rates for each possible combination of surveys and estimation methods. The root-mean-square-error and the bias were then used to summarize the accuracy and precision of the U5M model-based estimates.

The U5M posterior distribution was summarized by computing it’s mean and 95% credible intervals (CI) for each year between 1965 and 2013. 2013 was the last year with reliable data to allow prediction of U5M [41]. Using the posterior mean, the annual rate of reduction (ARR) was estimated at national and county level between 2000 and 2013 by assuming an exponential function in the change between the two-time points [42] (Additional file 3). The progress of the 47 counties in meeting the targets set during the World Summit for Children [3] between 1990 and 2000 was then assessed. Achievement of the MDG 4 [4] by 2015 was evaluated using projections computed via 2000–2013 AAR assuming the trajectory remained constant. Finally, the inequality in U5M across the counties between 1965 and 2013 was calculated by adapting the Palma’s inequality ratio [43] and computing the ratio as the average of U5M of 40% of the counties with the high U5M divided by the average U5M of 10% of counties with low U5M since 1965. The results were mapped in ArcMap 10·5 (ESRI Inc., Redlands, CA, USA).

## Results

**Fig. 2:**
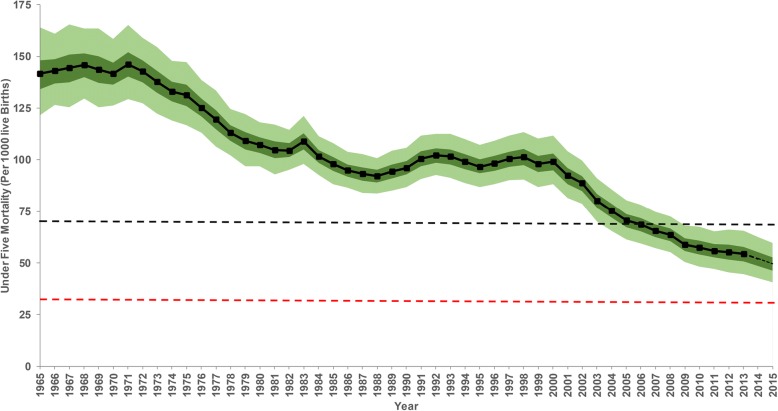
The national annual mean (black line), 2·5–97·5% (light green boundary) interquartile credibility range (ICR) and 25–75% ICR (dark green boundary) of all cause-under five mortality per 1000 live births (U5M) in Kenya between 1965 to 2015 computed using direct demographic methods. The 2014–2015 U5M rates were computed using the average annual rate of reduction between 2000 and 2013 and shown as a dotted line. The U5M reduction target for World Summit for Children by 2000 is shown by a black dotted horizontal line while the red indicates the Millennium Development Goal 4 target. The graphs of county specific mean U5M and the corresponding 2·5–97·5% ICR are presented in the appendix (Additional file 5)

The data assembled included ten sample household surveys and three population censuses carried out between 1989 and 2014 covering 460 county-years, 1·14 million households and 1·62 million women aged between 15 and 49 years (Table 1). The demographic methods and the spatio-temporal model allowed for 49 years of temporal predictions from 1965 to 2013 across all 47 counties. Overall, at the national level, there was a 61·6% reduction in the mean rate of U5M over 49 years from a rate of 141·7 (95% CI: 121·6–164·0) deaths per 1000 live births in 1965 to 54·5 (44·6–65·5) per 1000 live births in 2013 (Fig. 2). There were two periods of substantial decline over the 49 years from the early 1970s to late 1980s and early 2000 to 2013 with U5M appearing to stagnate and/or reverse during the 1990s. The highest ARR (4·5% [4·0–5·1]) was recorded between 2000 and 2013, 2·5 times faster relative to the rate of reduction in 1965–1988 (1·8% [1·5–2·0]). At a national level, the goals set out in the World Summit for Children were not achieved by 2000; the estimated U5M rate in 2000 was 99·3 (95% CI: 88·3–111·7) per 1000 live births against a target of 70 deaths per 1000 live births. The overall change between 1990 and 2000 was a 3·3% (Fig. 2) reduction, far short of the 33.3% target. Additionally, using the 2015 projected U5M rates, the two-thirds (66.67%) reduction target set as part of MDG 4 was not achieved nationally despite an overall reduction of 48·3% (43·6–53·1) between 1990 and 2015 (Fig. 2).

**Fig. 3:**
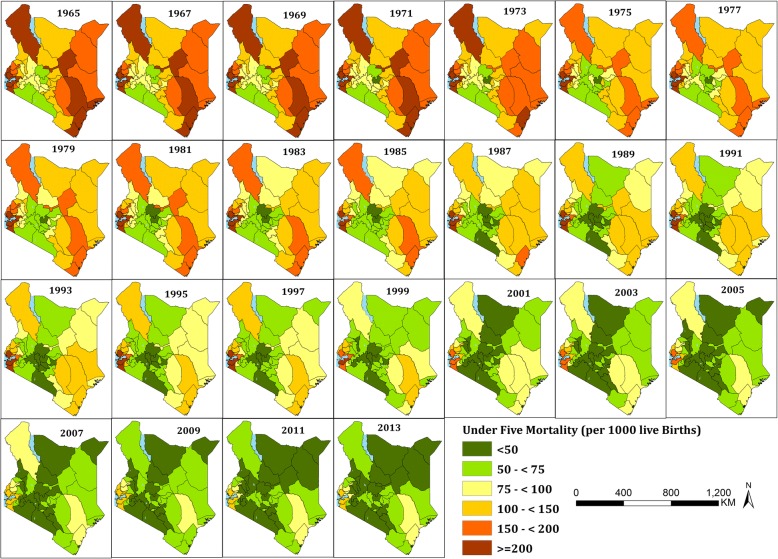
Mean under five mortality per 1000 live births (U5M) at each of the 47 counties of Kenya every two years between 1965 and 2013 classified into six classes of < 50 (dark green), 50 - < 75 (light green), 75 - < 100 (light yellow), 100 - < 150 (brown), 150 - < 200 (red), and ≥ 200 (dark brow)

**Fig. 4:**
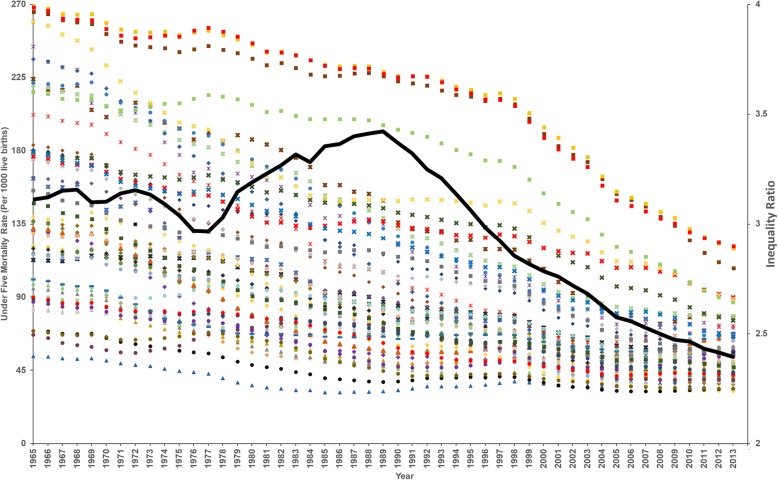
A scatter plot showing changes in mean under five mortality per 1000 live births (U5M) per county between 1965 and 2013. The provinces are differentiated by shapes; Coast (**ӿ**), North Eastern (♦), Eastern (**+**), Central (▲), Rift valley (●), Western (**x**), Nyanza (■) and Nairobi (**−**) and counties with colors (indexed in Table 2). The bold dark line shows inequality ratio calculated by dividing U5M of 40% of the counties with high U5M with the U5M of 10% of counties with low U5M between 1965 and 2013

These national estimates mask important sub-national differences in U5M rates over time (Fig. 3). Historically the highest mortality rates are those in the coastal areas, the arid and semi-arid areas around Lake Turkana and those around the Lake Victoria region (Fig. 3 and Fig. 4). The mean mortality rates in 1965 ranged from over 200 per 1000 live births in 11 counties to between 50 and 75 per 1000 live births in four counties. Overall, using 1965 as a baseline, by 2013, 23 counties had ≥60% decline in U5M rates, while a further 14 counties reduced U5M rates by at least a half (Additional file 4). All the counties except Nyeri and Nairobi had an overall reduction greater than a third (Additional file 4). By 2013, high mortality counties relative to the national average, continued to persist around Lake Victoria and the Tana river.

While the country did not achieve any of the goals set to reduce mortality rates in children below five years, there are some counties that achieved some of the goals (Table 2). Those that had an U5M rate of less than 70 per 1000 live births by 2000 included 24 counties (Mandera, Marsabit, Meru, Embu, Machakos, Makueni, Nyandarua, Nyeri, Kirinyaga, Murang’a, Kiambu, West Pokot, Samburu, Trans Nzoia, Uasin Gishu, Elgeyo Marakwet, Baringo, Laikipia, Nakuru, Narok, Kajiado, Kericho and Bomet). However, only two counties (Isiolo and Elgeyo Marakwet) had at least a third reduction in U5M rates between 1990 and 2000. No county in Western and Coast provinces achieved these targets compared to all counties in Central province and Nairobi that attained the targets set during the World Summit for Children to reduce U5M rates to ≤70 per 1000 live births by 2000 and/or a one-third reduction between 1990 and 2000 (Table 2).

**Table 2:**
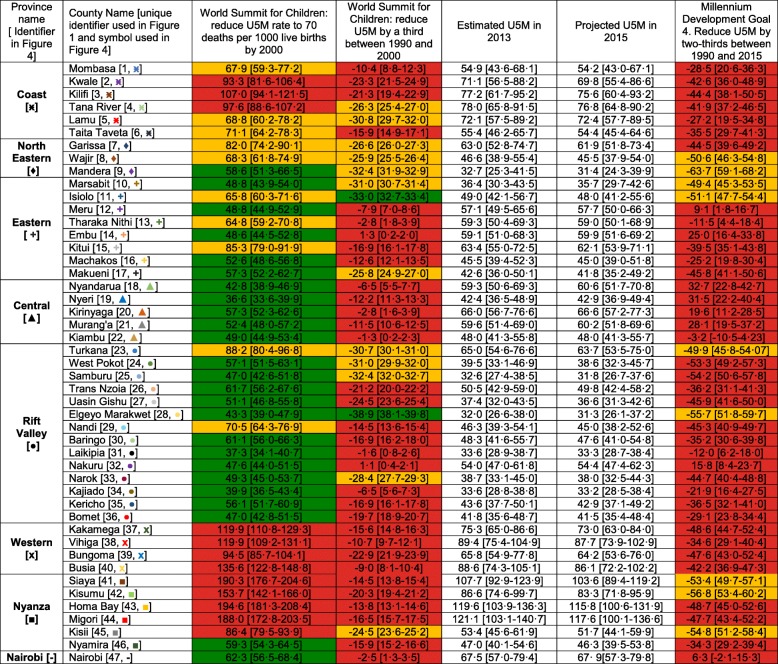
Progress of the 47 counties in meeting global under five mortality (U5M) reduction targets set during the World Summit for Children and the Millennium Development Goal 4 including the 2000, 2013 and 2015 projections U5M rates in Kenya

Counties that achieved the targets are shown in green, amber shows those that missed the target by a small margin (20%), while red indicates counties that missed the target with a large margin. The numbers (Fig. 1) and colors (Fig. 4) indicate the counties while the shapes (Fig. 4) indicate the 8 provinces of Kenya

No county achieved a two-thirds reduction in U5M rates between 1990 and 2015 (MDG 4) based on projections made to 2015, however, nine counties had reductions ≥50%, 34 reduced by at least 25% while four counties had reductions < 25% (Table 2). Whilst the national and sub-national MDG 4 was not achieved by 2015, more than half (25) of the counties reduced U5M by at least a third, while 30 had U5M rates below 70 per 1000 live births by 2015 achieving goals set during the World Summit for Children in 1990. Despite the goals being achieved in only some of the counties, 2015 projections show mean mortality rates remain relatively low (< 50 deaths per 1000 live births) in 21 counties (Nyeri, Kiambu, Trans Nzoia, Uasin Gishu, Wajir, Mandera, Marsabit, Isiolo, Machakos, Makueni, West Pokot, Samburu, Elgeyo Marakwet, Nandi, Baringo, Laikipia, Narok, Kajiado, Kericho, Bomet and Nyamira) (Table 2). Hence by 2015, no county had U5M rates of ≤25 per 1000 live births, the target for 2030 (SDG 3.2).

However, there were some notable exceptions during the MDG period (1990–2015), where U5M rates increased relative to the 1990 rates. Counties in the Central province (Murang’a, Kiambu, Kirinyaga and Nyeri) and the adjacent areas (Laikipia, Nakuru, Embu, Nairobi) had small upsurges between 1990 and 1995. By 2010–2013, the reversal had spread outwards to other counties in Central province, Coast (Kilifi and Mombasa), Eastern (Machakos, Makueni, Tharaka Nithi and Meru) and the south part of Rift Valley Province (Narok and Kajiado). The overall percentage increase in these eight counties ranged from 5·3 in Nairobi to 32·7 per 1000 live births in Nyandarua county between 1990 and 2015 (Table 2). The Additional file 5 has detailed county profiles and the corresponding 95% CIs graphs for each of the 47 counties.

On average, the gap between the highest and lowest recorded rates of U5M across Kenyan counties reduced by 58·6% (57·7–59·2), however, it was compounded by high magnitude of heterogeneity over time. By 2013, the ratio between the highest and lowest U5M rates was 3·8 (3·7–4·1) from a ratio of 5·0 (4·9–5·2) in 1965. The inequality index was high in the 1960s reduced marginally to 1970s then increased and remained high in the mid-1980s to mid-1990s. From mid-1990, the inequalities have declined dramatically through to 2013 (Fig. 4). Values recorded in 2013 shows that high inequality continues to exist notably in Western and Nyanza Provinces (Fig. 4).

## Discussion

In Kenya, shortly after independence, one in every seven Kenyan children born alive, died before the age of five, 49 years later, mortality has declined significantly, but remains high, with one in every 19 children not reaching their fifth birthday (Fig. 2). The progress towards improving child survival has been uneven within Kenya’s borders, with high rates of reduction in some areas and intractable slow progress in others (Fig. 3 and Fig. 4). Progress in meeting international goals aimed at reducing U5M has been less than optimal. Nationally, Kenya has not reached international goals set to reduce U5M rate by 2000 (World Summit for Children Goals) or 2015 (MDG 4) (Fig. 2), while only 25 counties met the 2000 goals, and none achieved the MDG 4 by 2015 (Table 2).

The declines in U5M started shortly after independence, through to 1988. Reductions coincide with efforts to establish policies to support economic growth, mitigate against high fertility rates and rapid population growth rates past independence in 1963 [44]. The stagnation of U5M rates and subsequent rise witnessed between 1988 and 2000 coincided with the increasing spread of the HIV epidemic and poverty [21, 45], low immunization coverage rates, poor quality of childcare [23], reintroduction of user fees [22], poor coverage of healthcare services, insufficient health workforce, a reduced focus on RMNCH [23, 25] and resurgent malaria epidemics driven by El Niño rainfall anomalies and escalating drug resistance [24, 46]. From 2000 through to 2013, influential strategic changes to support health financing and access to healthcare, more vaccines and other preventive measures, effective medicines for malaria control and scaleup of interventions to control and manage HIV all led to a dramatic change in effective health service access [21–27].

Understanding the relative contributions of the broad health sector changes to sub-national U5M reductions is difficult, however, what is clear is that the U5M transitions have not been equal between counties and over time. The counties with the highest levels of U5M in 1965–1970 experienced the greatest reductions while those with lower starting levels had smaller changes observed over the 49-year period. Children born in Homa Bay county were 5 times as likely to die before age 5 as children born in Nyeri county in 1965 and reduced to 3·8 times by 2013. The Western part of the country was the worst place to be born and remained the most disadvantaged through to 2013. These are areas characterized by high HIV prevalence [21] and intense malaria transmission [46]. Conversely high U5M in Northern part of the country, especially Turkana county, could be explained by harsh arid conditions leading to food insecurity and malnutrition [47].

Child mortality related inequalities have been documented across different socio-economic groupings (e.g. disparities in wealth, education etc.) as well as across geographic units [48]. Here the focus was on spatio-temporal inequalities between counties from 1965 to 2013 (Fig. 4) because spatial inequality links health outcomes to characteristics of a place that poverty or urban/rural taxonomies cannot highlight [49]. The cross-county inequalities were wide spread from 1965 to early 1970s and coincided with a phase when the Kenyan government was fighting poverty, ignorance and disease [13]. While U5M was decreasing during the 1980s, the existing inequalities were exacerbated and coincided with increasing HIV prevalence and rampant poverty across the country [45]. Conversely, inequalities started to decline in the early 1990s and linked to huge reductions in the differences between counties with low and high U5M rates and were sustained through to 2013. However, differences recorded in 2013 are still unacceptable and focus on reducing U5M in these high burden areas will narrow the gap.

Mortality rates in some of the counties of Central province appear to have stalled, stagnated, or reversed in most recent periods especially after 2003 (Additional file 5). These counties had low starting U5M level in 1965, experienced the smallest overall decline and despite the marginal increases in the recent years, they still have lower U5M levels relative to counties in Western areas of Kenya. The lower U5M rates might be explained by fertile lands supporting agriculture, economic and political stability, low prevalence of HIV and malaria. It is plausible that the residual deaths in these counties are those that occur during the first month of life accounting for about 45% of all under five deaths [50]. The continued declines in the other parts of Kenya are likely due to the deaths that occur outside the first month of life, however, further analysis is needed to establish the drivers’ behind the recent rises and or stagnation in Central Kenya.

Projections to 2015 were undertaken for each county (Table 2) using the 2000–2013 trajectory rates to track progress across the MDG period. However, no county achieved MDG 4, and given ambitious 2030 targets, additional targeted efforts are required. Projections to 2030 were not attempted due to concerns regarding long-term projections using insufficient data; the year for which the projections are made should lie closer to the actual year of the most recent data [51]. Finally, the heterogeneous patterns in U5M observed in time and space are likely to be ascribed to uneven distribution, access and utilization of interventions and resources. This requires further evaluations to better explain and quantify specific contributions of these differences to these observed trends.

## Conclusion

In conclusion, substantial progress in reducing U5M over time was witnessed subnationally, especially since 2000. However, the rates of progress were slow and unequal across counties and as a result, the progress towards achieving international child survival targets remains sub-optimal across counties and over time. Huge spatio-temporal inequalities continue to persist with substantial gaps between the best and worst performing counties. The estimates generated can be used as a baseline for long-term monitoring and tracking progress and to inform better and efficient allocation of the resources through policies and programs that allow for equitable progress within the decentralized health planning structure in Kenya.

## Additional files

Additional file 1: Data sources and assembly. (DOCX 20 kb)

Additional file 2: Methods of mapping district boundaries to county equivalent. (DOCX 21 kb)

Additional file 3: Demographic and spatio-temporal methods for estimating subnational under five mortality. (DOCX 89 kb)

Additional file 4: Mean under five mortality rate every ten years and the corresponding change between 1965 and 2013 by county in Kenya. (DOCX 21 kb)

Additional file 5: Graphs of county level mean under five mortality and the corresponding 2·5–97·5% interquartile credibility range in Kenya. (DOCX 1378 kb)

## Abbreviations

CBH: Complete Birth History
CD: Proportion of Children Dead
DHS: Demographic and Health Survey
MICS: Multiple Indicator Cluster Survey
RMNCH: maternal, newborn, and child health indicators
SBH: Summary Birth History
SSA: Sub-Saharan Africa
