# Supplementary material for: Sub national variation and inequalities in under-five mortality in Kenya since 1965
Source: BMC Public Health. 2019 Feb 4;19:146. doi: 10.1186/s12889-019-6474-1 (PMC6360661; doi:10.1186/s12889-019-6474-1)
Supplement: Supplementary file 1 — Data sources and assembly. (DOCX 20 kb) [file 12889_2019_6474_MOESM1_ESM.docx]

## Additional File 1 (AF1): Data Sources and assembly

Detailed data review resulted to six Demographic and Health Surveys (DHS), six Multiple Indicator Surveys (MICS) and three population censuses with birth history data to allow estimation of all cause under-five mortality (U5M) at county level in Kenya. The DHS program ([http://www.dhsprogram.com](http://www.dhsprogram.com/)) conducts nationally representative household surveys every five years to provide data on demographic and health indicators such as population demographics, fertility, family planning, maternal and child health, and infectious diseases. It uses a two-stage sampling design, with the primary sampling unit (cluster) drawn through a probability proportional to size sampling, from a national master frame with enumeration areas defined during the national census. At the second stage, approximately 25 households are selected by equal probability sampling from a household list in each selected cluster.

The United Nations Children's Fund (UNICEF), through MICS initiative (<http://mics.unicef.org/>), aids countries to collect data for monitoring the situation of children and women. The indicators collected relate to child health, education, child protection, water and sanitation among others. In addition, the Kenyan government through the national bureau of statistics (<https://www.knbs.or.ke/>) has been conducting decennial census. The Kenya census provides data on: size, composition and spatial distribution of the population; levels of fertility, mortality and migration rates; levels of education attained by the population; housing conditions and availability of household amenities among others.

Among the assembled data, two surveys were excluded, MICS 2009 and MICS 2013/14. The 2009 MICS was conducted in the informal settlements of in Mombasa, hence the estimated U5M would only be indicative of the rural population of the county. The 2013/2014 MICS conducted in three counties (Kakamega, Turkana and Bungoma) with under-5 deaths under-reported. It was excluded to guard against diluting the estimates computed from other data sources. AF1 Table1 shows data available per county over time, by survey.

AF1 Table 1: Data used to estimate U5M by county.

| **Province** | **County** | **Demographic and Health Survey (DHS** | | | | | | **Population Census** | | | **Multiple Indicator Cluster Survey** | | | |
| --- | --- | --- | --- | --- | --- | --- | --- | --- | --- | --- | --- | --- | --- | --- |
|  |  | **1989** | **1993** | **1998** | **2003** | **2008/09** | **2014** | **1989** | **1999** | **2009** | **2000** | **2007** | **2008** | **2011** |
| **Coast**  [ **ӿ**] | Mombasa [1, **ӿ**] | Y | Y | Y | Y | Y | Y | Y | Y | Y | Y | N | N | N |
|  | Kwale [2, **ӿ**] | Y | Y | Y | Y | Y | Y | Y | Y | Y | Y | N | N | N |
|  | Kilifi [3, **ӿ**] | Y | Y | Y | Y | Y | Y | Y | Y | Y | Y | N | N | N |
|  | Tana River [4, **ӿ**] | M | Y | M | Y | Y | Y | Y | Y | Y | Y | N | N | N |
|  | Lamu [5, **ӿ**] | M | Y | M | Y | Y | Y | Y | Y | Y | Y | N | N | N |
|  | Taita Taveta [6, **ӿ**] | Y | Y | Y | Y | Y | Y | Y | Y | Y | Y | N | N | N |
| **North Eastern** [ ♦] | Garissa [7, ♦] | N | N | N | Y | Y | Y | Y | Y | Y | Y | Y | N | N |
|  | Wajir [8, ♦] | N | N | N | Y | Y | Y | Y | Y | Y | Y | Y | N | N |
|  | Mandera [9, ♦] | N | N | N | Y | Y | Y | Y | Y | Y | Y | Y | N | N |
| **Eastern**  [**+**] | Marsabit [10, **+**] | N | N | N | Y | Y | Y | Y | Y | Y | M | N | Y | N |
|  | Isiolo [11, **+**] | N | N | N | Y | Y | Y | Y | Y | Y | Y | N | Y | N |
|  | Meru [12, **+**] | Y | Y | Y | Y | Y | Y | Y | Y | Y | Y | N | Y | N |
|  | Tharaka Nithi [13, **+**] | Y | Y | Y | Y | Y | Y | Y | Y | Y | Y | N | Y | N |
|  | Embu [14, **+**] | Y | Y | Y | Y | Y | Y | Y | Y | Y | Y | N | Y | N |
|  | Kitui [15, **+**] | Y | Y | Y | Y | Y | Y | Y | Y | Y | Y | N | Y | N |
|  | Machakos [16, **+**] | Y | Y | Y | Y | Y | Y | Y | Y | Y | Y | N | Y | N |
|  | Makueni [17, **+**] | Y | Y | Y | Y | Y | Y | Y | Y | Y | Y | N | Y | N |
| **Central**  [▲] | Nyandarua [18, ▲] | Y | Y | Y | Y | Y | Y | Y | Y | Y | Y | N | N | N |
|  | Nyeri [19, ▲] | Y | Y | Y | Y | Y | Y | Y | Y | Y | Y | N | N | N |
|  | Kirinyaga [20, ▲] | Y | Y | Y | Y | Y | Y | Y | Y | Y | Y | N | N | N |
|  | Murang’a [21, ▲] | Y | Y | Y | Y | Y | Y | Y | Y | Y | Y | N | N | N |
|  | Kiambu [22, ▲] | Y | Y | Y | Y | Y | Y | Y | Y | Y | Y | N | N | N |
| **Rift Valley**  [●] | Turkana [23, ●] | N | N | N | Y | Y | Y | Y | Y | Y | M | Y | N | N |
|  | West Pokot [24, ●] | Y | Y | Y | Y | Y | Y | Y | Y | Y | Y | N | N | N |
|  | Samburu [25, ●] | N | N | N | Y | Y | Y | Y | Y | Y | Y | N | N | N |
|  | Trans Nzoia [26, ●] | Y | Y | Y | Y | Y | Y | Y | Y | Y | Y | N | N | N |
|  | Uasin Gishu [27, ●] | Y | Y | Y | Y | Y | Y | Y | Y | Y | Y | N | N | N |
|  | Elgeyo Marakwet [28, ●] | Y | Y | Y | Y | Y | Y | Y | Y | Y | Y | N | N | N |
|  | Nandi [29, ●] | Y | Y | Y | Y | Y | Y | Y | Y | Y | Y | N | N | N |
|  | Baringo [30, ●] | Y | Y | Y | Y | Y | Y | Y | Y | Y | Y | N | N | N |
|  | Laikipia [31, ●] | Y | Y | Y | Y | Y | Y | Y | Y | Y | Y | N | N | N |
|  | Nakuru [32, ●] | Y | Y | Y | Y | Y | Y | Y | Y | Y | Y | N | N | N |
|  | Narok [33, ●] | Y | Y | Y | Y | Y | Y | Y | Y | Y | Y | N | N | N |
|  | Kajiado [34, ●] | Y | Y | Y | Y | Y | Y | Y | Y | Y | Y | N | N | N |
|  | Kericho [35, ●] | Y | Y | Y | Y | Y | Y | Y | Y | Y | Y | N | N | N |
|  | Bomet [36, ●] | Y | Y | Y | Y | Y | Y | Y | Y | Y | Y | N | N | N |
| **Western**  [**x**] | Kakamega [37, **x**] | Y | Y | Y | Y | Y | Y | Y | Y | Y | Y | N | N | N |
|  | Vihiga [38, **x**] | Y | Y | Y | Y | Y | Y | Y | Y | Y | Y | N | N | N |
|  | Bungoma [39, **x**] | Y | Y | Y | Y | Y | Y | Y | Y | Y | Y | N | N | N |
|  | Busia [40, **x**] | Y | Y | Y | Y | Y | Y | Y | Y | Y | Y | N | N | N |
| **Nyanza**  [■] | Siaya [41, ■] | Y | Y | Y | Y | Y | Y | Y | Y | Y | Y | N | N | Y |
|  | Kisumu [42, ■] | Y | Y | Y | Y | Y | Y | Y | Y | Y | Y | N | N | Y |
|  | Homa Bay [43, ■] | Y | Y | Y | Y | Y | Y | Y | Y | Y | Y | N | N | Y |
|  | Migori [44, ■] | Y | Y | Y | Y | Y | Y | Y | Y | Y | Y | N | N | Y |
|  | Kisii [45, ■] | Y | Y | Y | Y | Y | Y | Y | Y | Y | Y | N | N | Y |
|  | Nyamira [46, ■] | Y | Y | Y | Y | Y | Y | Y | Y | Y | Y | N | N | Y |
| **Nairobi**  [ **-**] | Nairobi [47, **-**] | Y | Y | Y | Y | Y | Y | Y | Y | Y | Y | N | N | N |

Y- Data collected; N- Data not collected; M surveyed counties, but data missing in the downloaded files. The symbols correspond to those used in Figure 1, Figure 2 and Table 2 in the manuscript.
