# Supplementary material for: Sub national variation and inequalities in under-five mortality in Kenya since 1965
Source: BMC Public Health. 2019 Feb 4;19:146. doi: 10.1186/s12889-019-6474-1 (PMC6360661; doi:10.1186/s12889-019-6474-1)
Supplement: Supplementary file 2 — Methods of mapping district boundaries to county equivalent. (DOCX 21 kb) [file 12889_2019_6474_MOESM2_ESM.docx]

## Additional File 2 (AF2): Mapping district boundaries to county equivalent

The county was used as the unit of analysis since it’s the resource allocation and decision-making unit in Kenya. Counties came into effect after the promulgation of the new constitution in 2010 and the 2013 general elections. Kenya became a decentralized state, with a central government and 47 semi-autonomous county governments [1, 2]. However, before counties became operational, districts were used as the administrative units [3, 4]. Consequently, household surveys and population census conducted before 2010, used the districts as the administrative units. Their number and size have changed over time ranging from 41 in 1989, 69 in 1999, 72 in 2003, to 198 in 2009. Therefore, all district boundaries were mapped to their corresponding county boundaries between 1989 and 2010. A unique list indicating the districts that either split or merged and how these matched to the existing counties was constructed.

Where available (DHS 2003 and 2008/09), cluster coordinates were used to map the equivalent county in ArcMap version 10·5 (ESRI Inc., Redlands, CA, USA). Otherwise, district names and codes were fast-tracked per survey type to figure out which district codes/names matched between similar surveys e.g. all the district codes/names of DHS 1989 were cross-walked with those of DHS 1993 followed by DHS 1998 then repeated for all MICS and census data. This was supported by information on how districts split over time [5–9], with a unique list generated after the exercise. The results were validated by extracting and digitizing district hardcopy maps from census, survey and related reports and overlaid them on the county boundaries in ArcMap version 10·5 (ESRI Inc., Redlands, CA, USA). Where ≥ 2 districts enclosed a county, data were aggregated and where two counties covered a single district, U5M was assumed to be equal in the two counties. The AF2 Table 1 summarizes the method used to map district to county boundary and technique used to re-distribute data across surveys.

AF2 Table 1: Mapping district boundary to equivalent county between 1989 and 2010

| **Survey and Year** | **Matching method** | **Observation and data re-allocation** |
| --- | --- | --- |
| DHS 2014 and MICS 2011 | County identifiers | County identifiers were provided with the microdata |
| DHS 2008/09 and 2003 | Coordinates | GPS cluster provided within the microdata of the two surveys were used to unambiguously remap district boundaries to their county equivalent |
| MICS 2007 | District code and name | All districts overlaid on county boundary seamlessly for MICS 2007 |
| DHS 1989, 1993, 1998 and Census 1989 | District code and name | One district covered two counties on six instances in each survey. U5M assumed to be similar in both the two counties enclosing a district |
| MICS 2000 and 2008 | District code and name | Two districts covered one county in 19 instances. Data in each of the two districts were aggregated to form a single county |
| Census 1999 | District code and name | Two districts enclosed one county in 16 cases, data aggregated to a single county  One district covered two counties in two cases. U5M assumed to be similar in both the two counties enclosing a district |
| Census 2009 | District code and name | 157 districts matched to 47 counties by aggerating data to form counties |

**DHS:** Demographic and Health Survey

**MICS:** Multiple Indicator Cluster Survey

**References**

1. Barasa EW, Manyara AM, Molyneux S, Tsofa B. Recentralization within decentralization: County hospital autonomy under devolution in Kenya. PLoS One. 2017;12:e0182440.

2. Government of Kenya. The Constitution of Kenya. 2010. http://kenyalaw.org/kl/index.php?id=398. Accessed 1 Jul 2017.

3. Oyaya CO, Rifkin SB. Health sector reforms in Kenya: An examination of district level planning. Health Policy (New York). 2003;64:113–27.

4. Mwabu G. Health care reform in Kenya: a review of the process. Health Policy (New York). 1995;32:245–55.

5. NCPD, CBS, Macro International Inc. Kenya Demographic and Health Survey 1993. 1994. https://www.dhsprogram.com/Publications/Publication-Search.cfm?ctry_id=20&c=Kenya&Country=Kenya&cn=Kenya. Accessed 17 Jan 2017.

6. NASCOP. 2007 Kenya AIDS Indicator Survey: Final Report. 2009. http://catalog.ihsn.org/index.php/catalog/429/download/30820. Accessed 30 Jun 2017.

7. KNBS. Census Cartography: The Kenyan Exeperince. Census Mapping and Use of Geographical Information Systems. 2007. https://unstats.un.org/unsd/demographic/meetings/egm/CensusMappingEGM07/docs/Kenya.pdf. Accessed 22 Feb 2018.

8. Government of Kenya. Kenya Service Availability and Readiness Assessment Mapping (SARAM). 2014;:341. http://apps.who.int/healthinfo/systems/datacatalog/index.php/catalog/4/download/39. Accessed 5 May 2017.

9. Law G. Districts of Kenya. 2013. http://www.statoids.com/yke.html. Accessed 9 Jan 2017.
