# Supplementary material for: Sub national variation and inequalities in under-five mortality in Kenya since 1965
Source: BMC Public Health. 2019 Feb 4;19:146. doi: 10.1186/s12889-019-6474-1 (PMC6360661; doi:10.1186/s12889-019-6474-1)
Supplement: Supplementary file 3 — Demographic and spatio-temporal methods for estimating subnational under five mortality. (DOCX 89 kb) [file 12889_2019_6474_MOESM3_ESM.docx]

## Additional File 3 (AF3): Modeling methods

## AF3 1: Estimation of under five mortality using demographic methods

Methods of estimating U5M using household surveys and population census data can be broadly classified into two categories based on the type of birth history available. The two types of birth histories include Complete Birth History (CBH) and Summary Birth History (SBH) and are collected through questions asked to women of childbearing age (15 to 49 years). The fundamental information collected for a CBH include the date of birth, whether the child is still alive, and if not, the age at death. The questions are asked chronologically starting with the first birth to the most recent [1–3]. A CBH incures more time and/or resources during the fieldwork, thus administered in a few sample household surveys [4].

SBH collects data on Children Ever Born alive (CEB) and Children Dead (CD) through questions posed in a variety of forms (AF3 Table 1 ) [5]. In principle only two responses are needed (CEB and CD) since mother’s age is a standard item collected in any survey and in some cases time since first birth or marriage. Therefore, fewer resources are incurred compared to CBH, consequently, SBH are included in almost all censuses, MICS and DHS [4, 6].

AF3 Table 1: SBH questions asked during a survey/census

| **Option 1** | 1. How many children, who were born alive, have you ever had? |
| --- | --- |
|  | 1. How many of those children have died? |
| **Option 2** | 1. How many children, who were born alive, have you ever had? |
|  | 1. How many are still alive? |
| **Option 3** | 1. How many living children do you have? |
|  | 1. How many children have you had who were born alive and later died? |
| **Option 4** | 1. How many children do you have who live with you? |
|  | 1. How many children do you have who live elsewhere? |
|  | 1. How many children have you had who were born alive and later died? |

One direct method and four indirect and independent methods were applied to CBH and SBH respectively to estimate U5M in each county by survey.

### AF3 1a: Direct method

Using CBH, the record of each child was expanded to include records for each month lived up to 59 months or to the point of death. An attribute was added in each child-month, to specify the survival status (dead or alive) of the kid at the end of the month. The child-months were allocated to six age-groups (0-1, 1–11, 12–23, 24–35, 36–47 and 48–59 months) based on mortality variation from 0 to 5 years. In addition, the child-months were assigned to two years’ time-periods with the estimated U5M assigned to the midpoint of each period [4, 7–11]. The monthly probability of survival for each age-group was computed as the ratio of deaths to the number of months lived in a particular age-group and time-period raised to a power equal to the number of months in the age group (Equation 1). These probabilities were multiplied to obtain the survival probability to age five and converted to U5M by subtracting the product of the survival probabilities from one (Equation 1) [8, 12].

$$q5_{t}=1-\left\{P_{t,mo.0}*\left(P_{t,mos.\text{ }1-11}\right)^{11}*\left(P_{t,\mathrm{yr}\text{ }1}\right)^{12}*\left(P_{t,\mathrm{yr}\text{ 2}}\right)^{12}*\left(P_{t,\mathrm{yr}\text{ 3}}\right)^{12}*\left(P_{t,\mathrm{yr}\text{ 4}}\right)^{12}\right\}-\text{Equation 1}$$

### AF3 1b Indirect methods of U5M estimation

The use of SBH to estimate U5M relies on the relationship between $\frac{CD}{CEB}$ abbreviated as $D\left( i \right)$ and the population-level child mortality for a cohort of women [13, 14]. The conversion of $D\left( i \right)$ to a probability of death before attaining a certain age x q(x) is influenced by the age pattern of fertility (constant *K(i*)) that determines the distribution of the children of a cohort of women by duration of exposure to the risk of dying. The approach has been refined over time by modifying *K(i)* [15, 16]*,* using the duration of marriage [15] or time since first birth as exposure [17] and localizing time to which the estimated U5M refer to [18]. These approaches have been used to estimate U5M [19] in absence of vital registration system data.

However, in 2010, these approaches were refined to allow estimation of U5M more robustly and accurately by addressing methodological limitations in the original approach. These adaptations include two cohort and two period methods indexed by either maternal age or time since birth as summarized in AF3 Table 2 [4]. ^T^hese four techniques were applied to quantify U5M rates where SBH data were available.

AF3 Table 2: Indirect Methods used to estimate U5M

|  | ***Period-Derived*** | ***Cohort-Derived*** |
| --- | --- | --- |
| **Maternal Age** | Maternal Age Period  **MAP** | Maternal Age Cohort  **MAC** |
| **Time Since First Birth** | Time Since First Birth Period  **TFBP** | Time Since First Birth Cohort  **TFBC** |

The cohort-derived methods groups data by cohorts defined by either the maternal age (MAC) or time since first Birth (TFBC). U5M rate was generated using a regression model (Equation 2) based on factors that affect probable timing of child deaths in the population [4].

Where (j) indexes a country, (k) a survey, (i) a cohort, CD is children dead, CEB is Children Ever Born while (*P*) are parity ratios. The coefficients $\left(\beta_{1i}-\beta_{4i}\right)$and country random effect (Uij) were generated using 166 DHS surveys pooled across 70 countries in SSA by relating CBH to SBH data [4].

The estimates generated from the 15–19 years cohort were discarded. This is because children born to these young mothers have higher risks of death compared to other age groups of women. This leads to higher than normal estimates of U5M [20]. The period-derived methods deal with these limitations by acknowledging that U5M for the most recent periods are due to both the mortality experiences of young mothers and older women [4]. In addition, the cohort methods generate U5M estimates for only 18 years preceding the time of the survey while period methods generate up to 25 years.

The Period methods calculates $D\left( i \right)$ for each year prior to the survey through the estimated distribution of births and deaths for mothers with a specific number of kids in a given age bracket. The distributions are indexed by either maternal age (MAP) or Time since first birth (TFBP) [4]. A regression model (Equation 3) was used to generate an U5M estimate for each year prior to survey. The coefficients were generated from the 166 DHS surveys pooled across 70 countries in SSA and further stratified into 5 broad regions e.g. SSA- East to capture regional heterogeneities [4].

$$Logit\left(5q0_{ijk}\right)=\beta^{0}_{t}+U_{tj}+\beta^{1}_{t}\log it\left(\frac{CD_{tjk}}{CEB_{tjk}}\right)+\varepsilon_{ijk}-\text{Equation 3}$$

To localize the estimated U5M to a specific time point (reference time), the CBH from the pooled data was used to determine the average time that deaths to women of age-group *i* occurred. The average time was then related to SBH data via a regression model (equation 4) to generate coefficients $\left(\beta_{1i}-\beta_{4i}\right)$ which were used to estimate reference time for SBH.

$$reference\text{ }time_{ijk}=\beta_{0i}+\beta_{1i}\left(\frac{CD_{ijk}}{CEB_{ijk}}\right)+\beta_{2i}CEB_{ijk}+\beta_{3i}\frac{P\left(15-19\right)_{jk}}{P\left(20-24\right)_{jk}}+\beta_{4i}\frac{P\left(20-24\right)_{jk}}{P\left(25-29\right)_{jk}}+\varepsilon_{ijk}-Equation\text{ 4}$$

The application of the five demographic techniques by county and survey resulted in multiple estimates of U5M in each county and year from 1965 to 2013 which required spatio-temporal smoothing.

## AF3 2: Spatio-temporal Smoothing

Spatial-temporal methods were used to obtain a single U5M estimate, per county and year while accounting for the small sample size, variability across the five demographic methods and surveys. More specifically, let $\left(Q_{ijkt}\right)$ denote the estimated U5M from the *i^th^* survey (n=13), based on method *j* (n=5) for the *k^th^* county (n=47) in year *t* (n=49), 1965-2013. A model (Equation 5), where $S_{kt}$is a spatio-temporal Gaussian process and $Z_{ijkt}$ is Gaussian noise.

$$\log\left\{\frac{Q_{ijkt}}{1-Q_{ijkt}}\right\}=\alpha+S_{kt}+Z_{ijkt}-Equation\text{ 5}$$

To predict $S_{kt}$, we borrow strength of information across all surveys, methods, counties and years. We model $S_{kt}$as a spatio-temporal Gaussian process with mean 0 and covariance function $\Sigma=\sigma^{2}\left[R_{S}\otimes R_{T}\right]$ where is the Kronecker product while R_S_ and R_T_ are the spatial and temporal correlation matrices, respectively. R_S_ is modelled using a conditionally autoregressive (CAR) process with correlation parameter $\rho$ such that (Equation 6)

$$\text{ S}_{kt}\left|\text{S}_{k't}\right.:k^{j}\in\delta_{k}\sim N\left(\rho\sum_{k^{j}\in\delta_{k}} \text{S}_{k't'}\frac{1}{\left|\delta_{k}\right|}\right)-Equation\text{ 6}$$

where $\delta_{k}$ is the set of counties that neighbours to the *k^th^* county and $\left|\delta_{k}\right|$ is their total number; we model R_T_ as an autoregressive process of the first order with a parameter of temporal correlation . The $Z_{ijkt}$ represent the residual sampling error which we assume to be a set independent of Gaussian variables with mean zero and variance $w_{i,k}^{2}\tau_{j}^{2}$ varying across surveys, $w_{i}$ is a weight equal to the log n_ik_, n is the number of interviewed women in the *i^th^* survey and *k^th^* county and $\tau_{i}^{2}$ expresses the variability of the estimator from the j^-th^ method. The model was fit using Bayesian inference, using the following independent diffuse priors.

$\beta_{i}$, *i*, for *i* = 0; 1, each follows a Gaussian distribution with mean 0 and variance 10^5^.

$\sigma^{2}$and $\tau_{i}^{2}$ for *i* = 1..N, have log-Gaussian priors with expectation is 1 and variance is 25.

$\rho$ and each follows a Uniform distribution in the unit interval [0, 1].

To fit the model, a Markov Chain Monte Carlo algorithm was developed in the R software (Version 3·4·1) and generated 10,000 predictive samples of smoothed U5M rates by county and year. To reduce the between-samples correlation, the algorithm was iterated for 110,000 times and retained every 10th sample after a burn-in of 10,000 samples. The U5M posterior distribution was summarized by computing the mean, and 95% credible intervals. The results were exported and mapped in ArcMap 10·5 (ESRI Inc., Redlands, CA, USA).

## AF3 3: Estimation of the average annual rate (AAR) of under-five mortality decline:

The AAR 2000 between 2000 and 2013 was calculated using U5M rates in 2000 and 2013, with an assumption that the annual rate of decline over this period was constant at each of the 47 counties and nationally [21, 22]. The change in U5M rates was assumed to take an exponential function similar to a compound annual rate of decline. Specifically, for any given year *t (2000)*, if the U5M rate is known to be **U5M_(t=2000)_**, and the AAR is constantly *AAR%*, then the U5M rates in *t+n (2013)* denoted as **U5M_(2000+n=2013)_** was calculated as:

$AAR=\left(\frac{U5M_{\left(2013\right)}}{U5M_{\left(2000\right)}}\right)^{\left(\frac{1}{\left(2013-2000\right)}\right)}-1$ Equation 7

## AF3 4: Model Validation

To assess the predictive performance of the model, a cross-validation procedure was used with a randomly selected hold-out sample containing 10% of the observed U5M rates for each possible combination of surveys and estimation methods. The root-mean-square-error and the bias were then used to summarize the accuracy and precision of the U5M model-based estimates. The model predictive performance of the sub-national temporal U5M showed a fairly high correlation with the observed values of 0·6, a bias of only 0·5%, and a root mean square of 0·08 indicating a small magnitude of error between the smoothed values and the demographic estimates.

**References**

1. Hill K, Brady E, Zimmerman L, Montana L, Silva R, Amouzou A. Monitoring change in child mortality through household surveys. PLoS One. 2015;10:e0137713. doi:10.1371/journal.pone.0137713.

2. United Nations. Step-by-Step Guide to the Estimation of Child Mortality. New York; 1990.

3. Curtis S. Assessment of the quality of data used for direct estimation of infant and child mortality in DHS-II surveys. Occasional Papers. 1995. http://www.getcited.org/pub/100178189.

4. Rajaratnam JK, Tran LN, Lopez AD, Murray CJLL. Measuring under-five mortality: Validation of new low-cost methods. PLoS Med. 2010;7:e1000253.

5. United Nations. Principles and Recommendations for a Vital Statistics System. Statistical Papers, Series M No. 19/Rev.3. 2014;:1–224.

6. Hill K, Lopez AD, Shibuya K, Jha P. Interim measures for meeting needs for health sector data: births, deaths, and causes of death. Lancet. 2007;370:1726–35.

7. Pedersen J, Liu J. Child Mortality Estimation: Appropriate Time Periods for Child Mortality Estimates from Full Birth Histories. PLoS Med. 2012;9:e1001289.

8. Dwyer-Lindgren L, Gakidou E, Flaxman A, Wang H. Error and bias in under-5 mortality estimates derived from birth histories with small sample sizes. Popul Health Metr. 2013;11:1–17.

9. Bauze AE, Tran LN, Nguyen KH, Firth S, Jimenez-Soto E, Dwyer-Lindgren L, et al. Equity and geography: The case of child mortality in Papua new Guinea. PLoS One. 2012;7:e37861.

10. King G, Tomz M, Wittenberg J. Making the Most of Statistical Analyses: Improving Interpretation and Presentation. Am J Pol Sci. 2000;44:347. doi:10.2307/2669316.

11. Bermejo R, Firth S, Hodge A, Jimenez-Soto E, Zeck W. Overcoming stagnation in the levels and distribution of child mortality: The case of the Philippines. PLoS One. 2015;10:e0139458.

12. Dwyer-Lindgren L, Kakungu F, Hangoma P, Ng M, Wang H, Flaxman AD, et al. Estimation of district-level under-5 mortality in Zambia using birth history data, 1980-2010. Spat Spatiotemporal Epidemiol. 2014;11:89–107. doi:10.1016/j.sste.2014.09.002.

13. Myburgh C. Estimating the fertility and mortality of African populations from the total number of children ever born and the number of these still living. Popul Stud (NY). 1956;10:193–206.

14. Brass W. Uses of census or survey data for the estimation of vital rates. In: African Seminar on Vital Statistics. Addis Ababa: United Nations Economic and Social Council; 1964. p. 1–27. http://repository.uneca.org/handle/10855/9560. Accessed 17 Feb 2017.

15. Sullivan JM. Models for the estimation of the probability of dying between birth and exact ages of early childhood. Popul Stud (NY). 1972;26:79–97.

16. Trussell TJ. A re-estimation of the multiplying factors for the Brass technique for determining childhood survivorship rates. Popul Stud (NY). 1975;29 February 2015:97–107.

17. Hill K, Figueroa M-E. Child Mortality Estimation by Time Since First Birth. In: Brass W, Zaba B, Blacker J, editors. Brass Tacks: Essays in Medical Demography. 2001. p. 1–301.

18. Feeney G. Estimating infant mortality trends from child survivorship data†. Popul Stud (NY). 1980;34:109–28.

19. Storeygard A, Balk D, Levy M, Deane G. The Global Distribution of Infant Mortality: A subnational spatial view. Popul Sp Place. 2008;14:209–29.

20. Ewbank DC. The Sources of Error in Brass’s Method for Estimating Child Survival: The Case of Bangladesh. Popul Stud (NY). 1982;36:459–74.

21. UNICEF. How to calculate Average Annual Rate of Reduction ( AARR ) of Underweight Prevalance. 2007. https://data.unicef.org/wp-content/uploads/2015/12/Technical_Note_AARR_185.pdf. Accessed 25 Jul 2017.

22. Amouzou A, Habi O, Bensaïd K. Reduction in child mortality in Niger: A Countdown to 2015 country case study. Lancet. 2012;380:1169–78.
