# Supplementary material for: Sub national variation and inequalities in under-five mortality in Kenya since 1965
Source: BMC Public Health. 2019 Feb 4;19:146. doi: 10.1186/s12889-019-6474-1 (PMC6360661; doi:10.1186/s12889-019-6474-1)
Supplement: Supplementary file 4 — Mean under five mortality rate every ten years and the corresponding change between 1965 and 2013 by county in Kenya. (DOCX 21 kb) [file 12889_2019_6474_MOESM4_ESM.docx]

**Additional File 4 (AF4)**

**AF4 Table** 1: U5M rates every ten years between 1965 and 2013 and the average percentage in U5M between 1965 and 2013 in each county of Kenya. Dark green shows reduction ≥ 65%, light green shows reductions between ≥ 45% and < 65% while orange show reductions less than 45%

| **County** | **1965** | **1975** | **1985** | **1995** | **2005** | **2013** | 1965-2013 % |
| --- | --- | --- | --- | --- | --- | --- | --- |
| **Mombasa** | 162·8 [130·9-198] | 126·4 [110·6-144·0] | 90·5 [81·0-100·1] | 77·5 [69·3-86·6] | 49·0 [42·1-57·0] | 54·9 [43·6-68·1] | -66·2 |
| **Kwale** | 243·7 [201·8-290·3] | 177·5 [156·6-200·3] | 138·5 [124·6-153·1] | 108·4 [96·7-121·2] | 73·0 [62·7-84·4] | 71·1 [56·5-88·2] | -70·8 |
| **Kilifi** | 223·8 [184·5-267·8] | 193·6 [170·6-217·8] | 158·8 [143·4-175·1] | 125·2 [112·3-139·0] | 77·8 [67·1-89·3] | 77·2 [61·7-95·2] | -65·5 |
| **Tana River** | 220·1 [186·4-256·3] | 186·6 [167·8-207·0] | 152·4 [139·8-166·0] | 115·6 [105·5-125·8] | 83·9 [75·4-93·0] | 78·0 [65·8-91·5] | -64·5 |
| **Lamu** | 202·1 [162·6-247·4] | 165·3 [143-189·1] | 112·0 [99·0-125·6] | 84·6 [75·0-94·9] | 62·2 [54·0-71·3] | 72·1 [57·5-89·2] | -64·3 |
| **Taita Taveta** | 112·6 [95·6-131·3] | 113·5 [102·9-125·1] | 93·1 [85·4-101·1] | 80·4 [73·4-87·6] | 60·1 [53·7-67·1] | 55· 4[46·2-65·7] | -50·8 |
| **Garissa** | 180·6 [150·4-214·7] | 149·6 [132·4-168·5] | 124·7 [113·3-136·4] | 98·4 [89·8-107·3] | 67·9 [60·9-75·4] | 63·0 [52·8-74·7] | -65·1 |
| **Wajir** | 183·4 [152·8-217·7] | 143·8 [126·8-162·3] | 107·7 [97·8-118·2] | 80·2 [73·1-88·0] | 58·9 [52·9-65·4] | 46·6 [38·9-55·4] | -74·5 |
| **Mandera** | 163·4 [125·3-208·4] | 126·4 [105·9-149·7] | 94·2 [82·6-106·4] | 77·0 [68·1-86·6] | 49·6 [42·8-57·1] | 32·7 [25·3-41·5] | -80·0 |
| **Marsabit** | 136·8 [112·9-163·1] | 116·3 [102·0-131·8] | 88·6 [80·1-97·8] | 60·1 [54·5-66·4] | 41·5 [37·0-46·3] | 36·4 [30·3-43·5] | -73·4 |
| **Isiolo** | 236·1 [204·6-270] | 182·6 [164·8-201·5] | 125·0 [115-135·1] | 80·3 [74·0-87·1] | 54·4 [49·3-59·9] | 49·0 [42·1-56·7] | -79·2 |
| **Meru** | 130·6 [114·6-147·8] | 90·2 [82·9-97·8] | 57·0 [53·0-61·1] | 51·5 [47·8-55·3] | 45·6 [41·6-49·8] | 57·1 [49·5-65·6] | -56·2 |
| **Tharaka Nithi** | 136·1 [118-155·6] | 98·7 [90·0-107·7] | 68·3 [63·1-73·8] | 66·8 [61·5-72·3] | 56·5 [50·9-62·5] | 59·3 [50·4-69·3] | -56·4 |
| **Embu** | 101·2 [88·0-116·0] | 71·6 [65·2-78·3] | 50·1 [46·4-53·9] | 50·0 [46·2-53·9] | 48·4 [44·0-53·1] | 59·1 [51-68·3] | -41·6 |
| **Kitui** | 173·9 [153·7-195·3] | 141·2 [130·4-152·3] | 110·5 [103·3-117·9] | 97·9 [91·2-104·9] | 70·4 [64·4-76·8] | 63·4 [55-72·5] | -63·5 |
| **Machakos** | 121·4 [106·9-136·9] | 101·4 [93·6-109·5] | 69·6 [64·9-74·5] | 58·7 [54·7-63·0] | 45·0 [41·0-49·1] | 45·5 [39·4-52·3] | -62·5 |
| **Makueni** | 119·7 [102·9-138·3] | 106·1 [96·6-116·1] | 84·1 [77·7-90·7] | 73·2 [67·2-79·5] | 45·3 [40·7-50·0] | 42·6 [36-50·1] | -64·3 |
| **Nyandarua** | 98·1 [83·9-113·2] | 76·1 [69·2-83·4] | 52·7 [48·5-57·2] | 42·4 [38·9-46·1] | 45·4 [40·8-50·3] | 59·3 [50·6-69·3] | -39·5 |
| **Nyeri** | 53·7 [46·3-61·8] | 44·5 [40·5-48·7] | 31·2 [28·9-33·7] | 35·3 [32·7-38·2] | 35·1 [31·8-38·7] | 42·4 [36·5-48·9] | -20·8 |
| **Kirinyaga** | 132·3 [115·2-150·9] | 100·5 [92·0-109·5] | 62·5 [57·8-67·5] | 56·9 [52·4-61·6] | 54·2 [49·0-59·8] | 66·0 [56·7-76·6] | -50·1 |
| **Murang'a** | 95·1 [82·3-109·1] | 71·0 [64·8-77·5] | 50·3 [46·7-54·2] | 48·3 [44·5-52·2] | 54·0 [49·0-59·4] | 59·6 [51·4-69] | -37·3 |
| **Kiambu** | 82·6 [71·2-94·8] | 68·3 [62·3-74·7] | 54·0 [50·1-58·3] | 50·8 [46·9-54·8] | 44·3 [40·1-48·8] | 48·0 [41·3-55·8] | -41·8 |
| **Turkana** | 221·7 [186·5-259·8] | 193·6 [172·3-215·7] | 150·3 [137·4-164·1] | 108·8 [99·7-118·9] | 78·6 [70·9-86·8] | 65·0 [54·6-76·6] | -70·6 |
| **West Pokot** | 116·6 [98·0-136·7] | 102·6 [92·5-113·4] | 86·0 [78·7-93·7] | 72·8 [66·5-79·5] | 47·7 [42·6-53·3] | 39·5 [33·1-46·9] | -66·0 |
| **Samburu** | 116·0 [96·9-137·4] | 101·7 [90·0-114] | 79·4 [72·2-87·0] | 60·2 [54·9-65·7] | 38·3 [34·2-42·7] | 32·6 [27·4-38·5] | -71·9 |
| **Trans Nzoia** | 129·0 [111·1-148·7] | 117·1 [106·9-128·4] | 92·8 [85·6-100·2] | 70·9 [65·3-76·9] | 51·2 [45·9-56·7] | 50·5 [42·9-59] | -60·8 |
| **Uasin Gishu** | 81·8 [70·4-94·6] | 78·8 [71·9-86·2] | 71·7 [66·3-77·3] | 62·1 [57·2-67·2] | 41·8 [37·9-46·1] | 37·4 [32-43·5] | -54·3 |
| **E. Marakwet** | 139·1 [117·9-161·9] | 124·2 [112-136·9] | 81·9 [74·8-89] | 58·0 [52·8-63·5] | 36·0 [32·1-40·2] | 32·0 [26·6-38] | -77·0 |
| **Nandi** | 101·4 [87·4-117·2] | 91·2 [82·8-99·8] | 88·7 [81·9-95·8] | 79·1 [72·8-85·7] | 57·2 [51·6-63·2] | 46·3 [39·3-54·1] | -54·3 |
| **Baringo** | 90·6 [78·5-103·6] | 72·8 [66·5-79·5] | 71·6 [66·3-77] | 71·3 [66-76·8] | 52·4 [47·6-57·5] | 48·3 [41·6-55·7] | -46·6 |
| **Laikipia** | 69·2 [59·3-80·0] | 56·8 [51·6-62·4] | 40·0 [37-43·3] | 40·6 [37·4-44] | 32·2 [29·1-35·6] | 33·6[28·9-38·7] | -51·4 |
| **Nakuru** | 86·8 [76·0-98·7] | 74·5 [68·5-80·7] | 56·7 [52·8-60·8] | 47·9 [44·6-51·5] | 45·3 [41·3-49·5] | 54·0 [47-61·8] | -37·8 |
| **Narok** | 66·6 [56·7-77·8] | 59·9 [54·4-65·7] | 74·0 [68·4-80·0] | 60·7 [56-65·8] | 39·6 [35·8-43·6] | 38·7 [33·1-45] | -41·9 |
| **Kajiado** | 69·1 [59·7-79·5] | 65·6 [59·9-71·8] | 51·7 [47·9-55·9] | 42·3 [39·1-45·8] | 35·3 [32·0-38·8] | 33·6 [28·8-38·8] | -51·3 |
| **Kericho** | 88·6 [77·5-100·5] | 79·0 [72·6-85·8] | 73·4 [68·5-78·8] | 63·2 [58·8-68] | 47·2 [42·8-51·8] | 43·6 [37·7-50·1] | -50·7 |
| **Bomet** | 89·6 [76·9-103·3] | 79·9 [72·8-87·6] | 69·8 [64·5-75·3] | 50·8 [46·6-55·2] | 41·5 [37·3-45·9] | 41·8 [35·6-48·7] | -53·3 |
| **Kakamega** | 179·5 [158·5-202·5] | 162·1 [149·8-175] | 148·1 [138·3-158·2] | 134·9 [125·4-144·5] | 96·5 [88·0-105·7] | 75·3 [65-86·6] | -58·0 |
| **Vihiga** | 176·2 [152·4-201·3] | 153·9 [140·8-167·9] | 133·9 [123·7-144·5] | 127·7 [117·3-138·4] | 107·8 [96·7-119·2] | 89·4 [75·4-104·9] | -49·2 |
| **Bungoma** | 179·2 [153·0-208·4] | 153·6 [139·5-168·6] | 132·3 [121·6-143·4] | 111·1 [101·6-121·1] | 79·7 [71·2-88·9] | 65·8 [54·9-77·8] | -63·3 |
| **Busia** | 259·2 [225·0-295·7] | 197·1 [179·5-216·1] | 150·8 [138·7-163·4] | 148·1 [135·7-161·4] | 112·0 [100·1-124·7] | 88·6 [74·3-105·1] | -65·8 |
| **Siaya** | 265·5 [234·3-298·2] | 240·8 [222·6-259·6] | 226·0 [211·3-241·3] | 213·2 [199·4-227·3] | 153·6 [140·9-166·7] | 107·7 [92·9-123·9] | -59·4 |
| **Kisumu** | 216·2 [189·6-244·3] | 209·0 [193·2-225·5] | 199·3 [186·1-213] | 177·9 [165·5-190·3] | 120·6 [110·3-131·3] | 86·6 [74·6-99·7] | -59·9 |
| **Homa Bay** | 268·7 [239·2-300·3] | 251·0 [233·5-269·2] | 233·0 [218·7-247·6] | 217·4 [203·9-231·4] | 155·0 [143·0-167·9] | 119·6 [103·9-136·3] | -55·5 |
| **Migori** | 268·0 [234·2-304·4] | 250·2 [229·6-271·5] | 232·2 [215·9-249·0] | 215·4 [199·9-231·6] | 149·5 [135·8-164·0] | 121·1 [103·1-140·7] | -54·8 |
| **Kisii** | 155·4 [135·1-178·0] | 130 [119·2-141·5] | 117·4 [108·9-126·2] | 102·9 [95·2-110·8] | 65·4 [59·5-71·6] | 53·4 [45·6-61·9] | -65·6 |
| **Nyamira** | 148·0 [128·3-169·5] | 108·9 [99·6-118·8] | 82·2 [76·2-88·7] | 62·2 [57·2-67·3] | 51·5 [46·8-56·7] | 47·0 [40·1-54·6] | -68·2 |
| **Nairobi** | 100·4 [85·2-117·0] | 74·2 [67·0-82·0] | 65·2 [59·9-70·8] | 67·4 [61·7-73·4] | 56·1 [50·3-62·6] | 67·5 [57-79·4] | -32·7 |
