# Supplementary material for: Sub national variation and inequalities in under-five mortality in Kenya since 1965
Source: BMC Public Health. 2019 Feb 4;19:146. doi: 10.1186/s12889-019-6474-1 (PMC6360661; doi:10.1186/s12889-019-6474-1)

## Additional File 5 (AF5): County U5M Profiles

The annual mean (black line) and 2·5–97·5% (light grey boundary) interquartile credibility range (ICR) of all cause-under five mortality per 1000 live births (U5M) in Kenya between 1965 to 2013 per county. The graphs have been ordered by the eight provinces of Kenya.

#### Coast Province


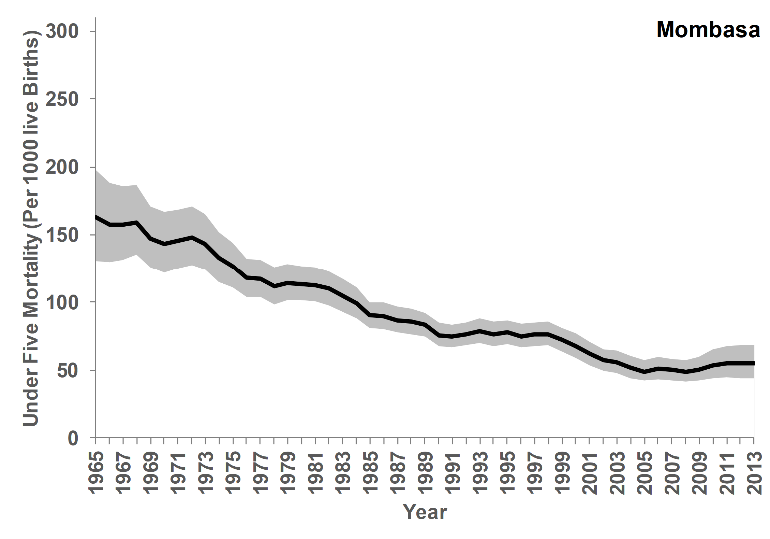

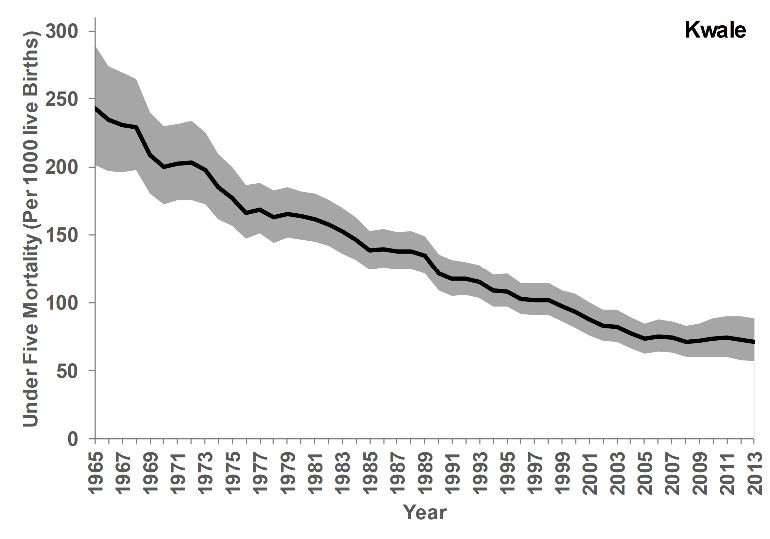


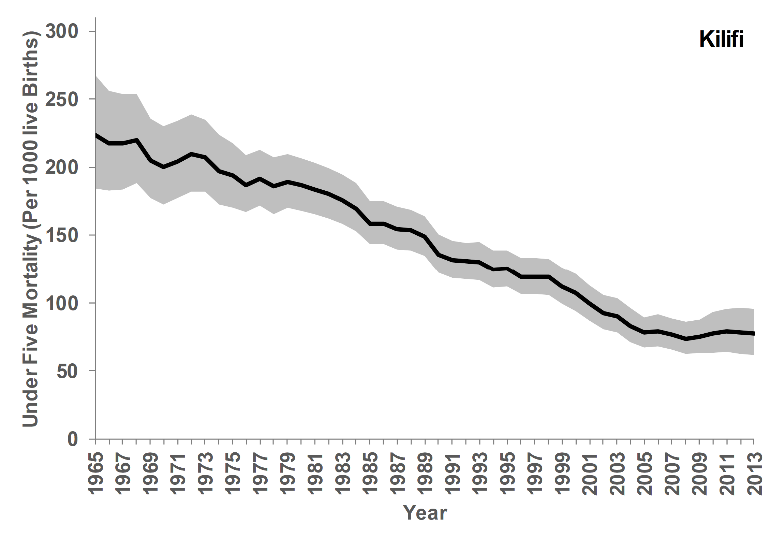

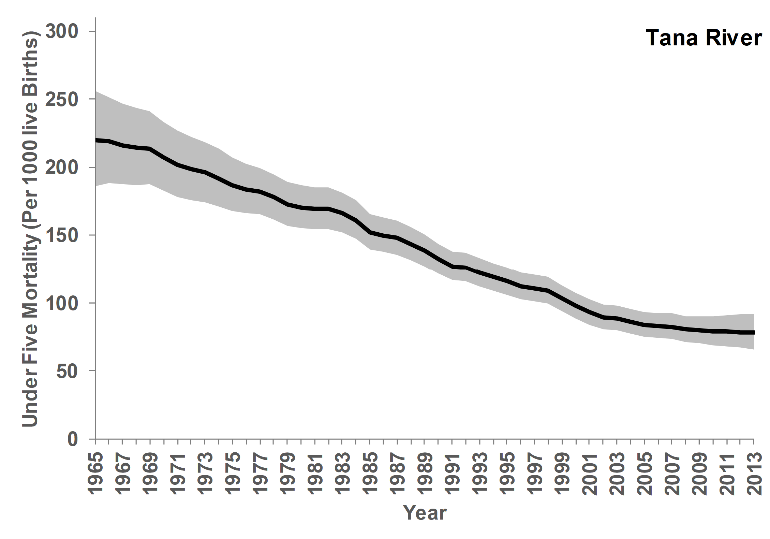


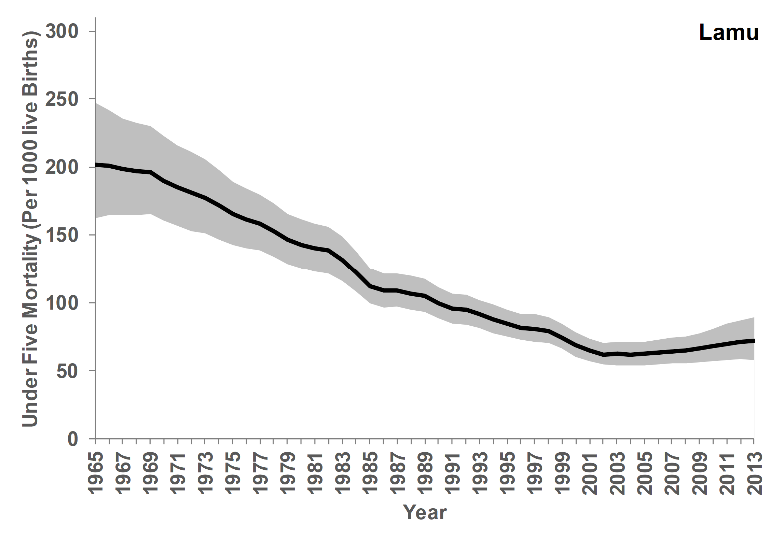

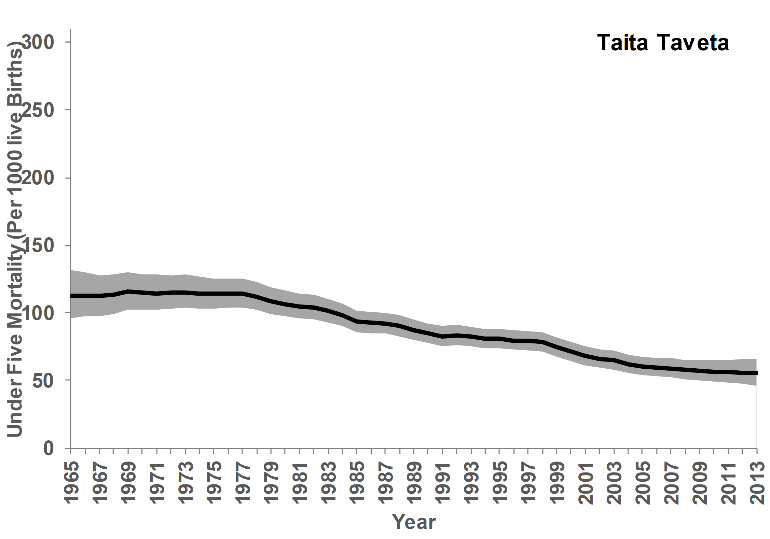


#### North Eastern Province


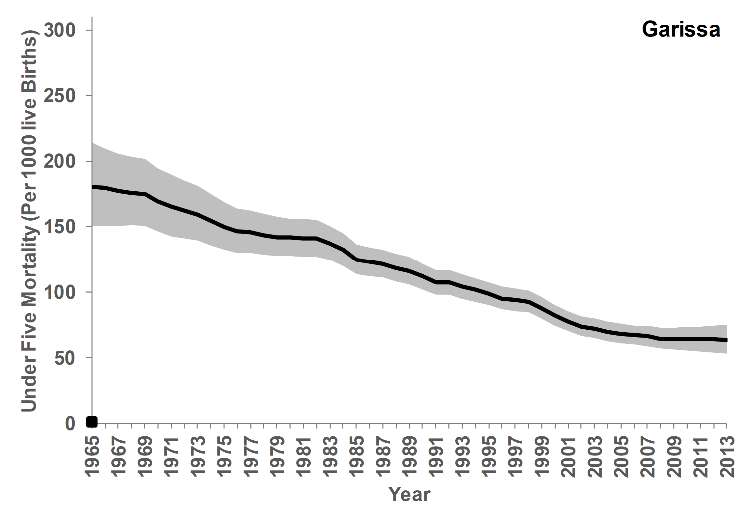

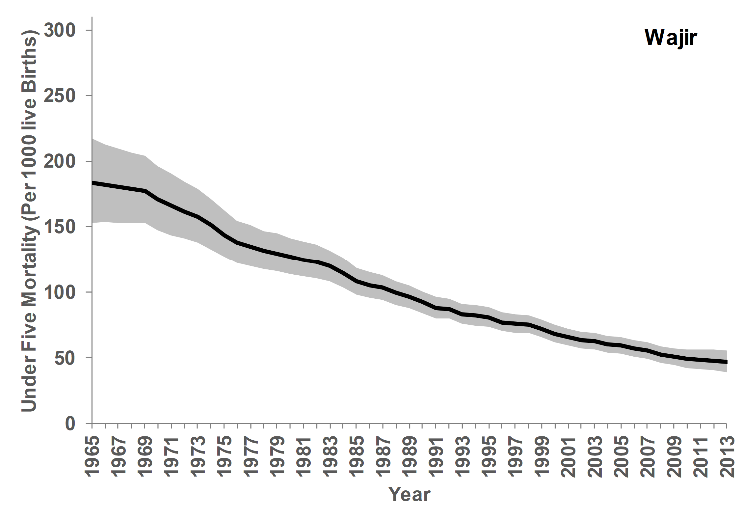


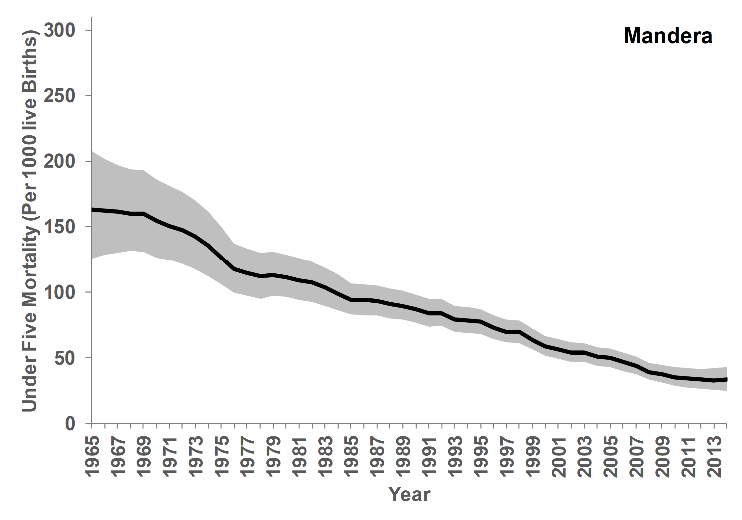


#### Eastern Province


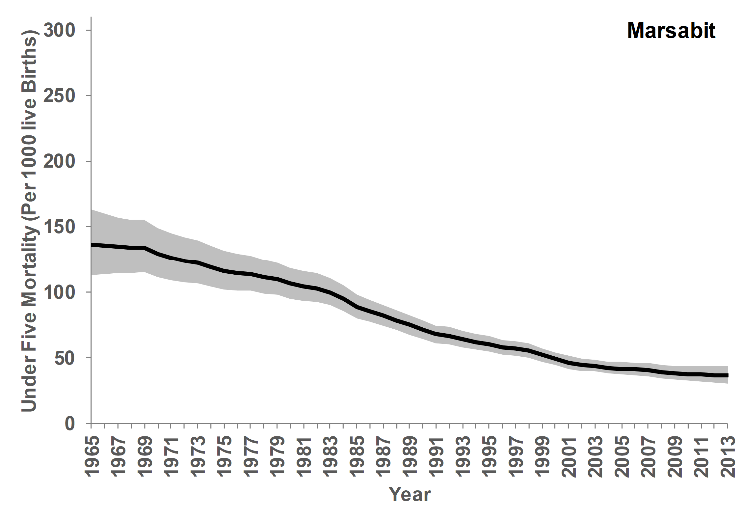

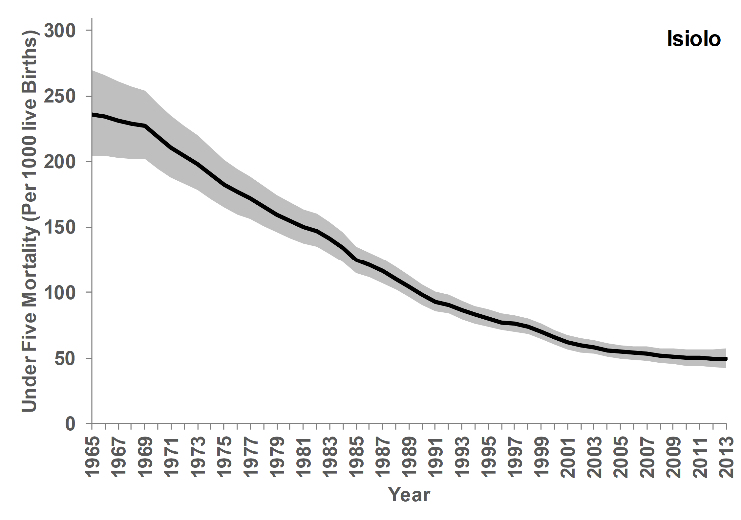


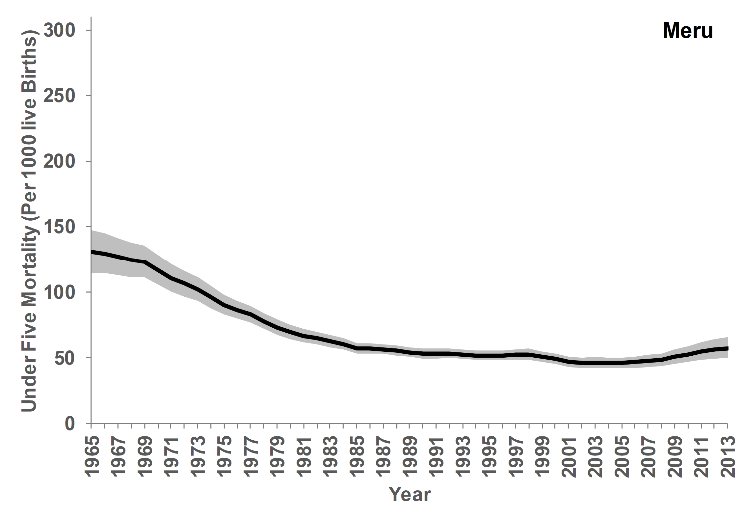

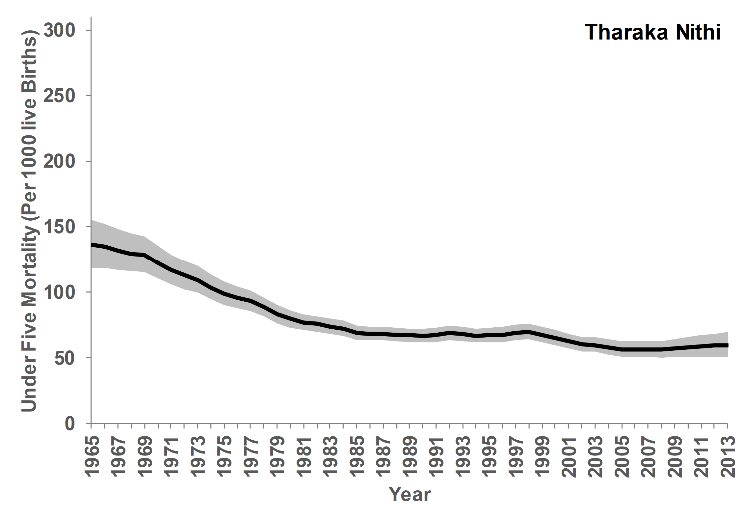


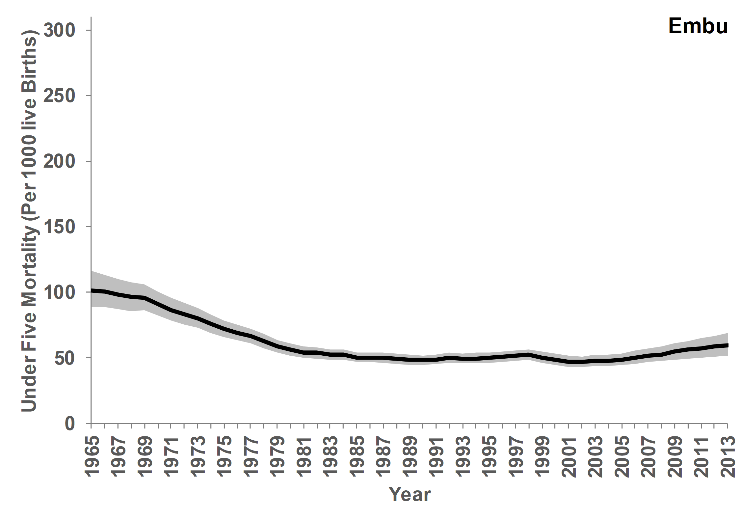

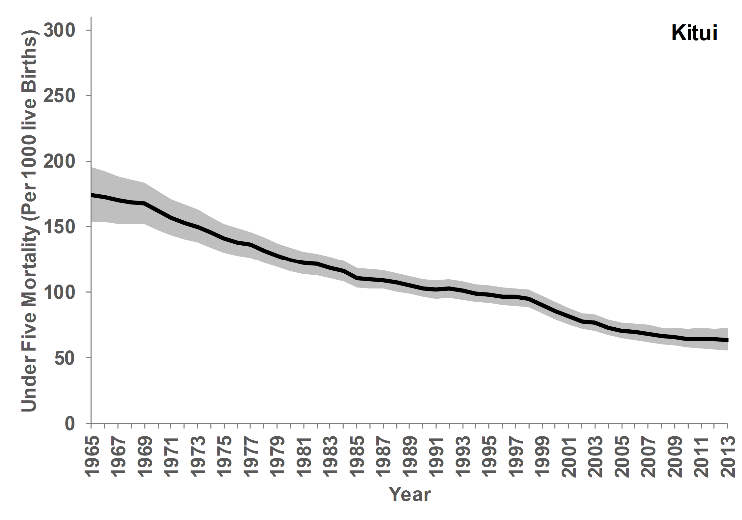


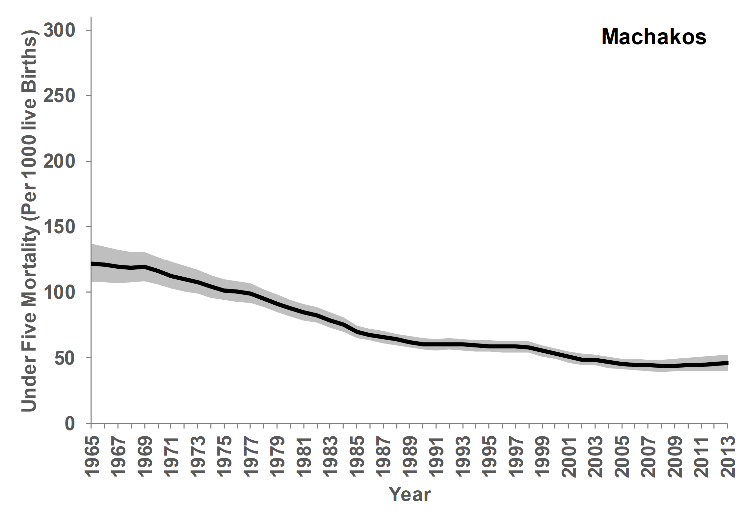

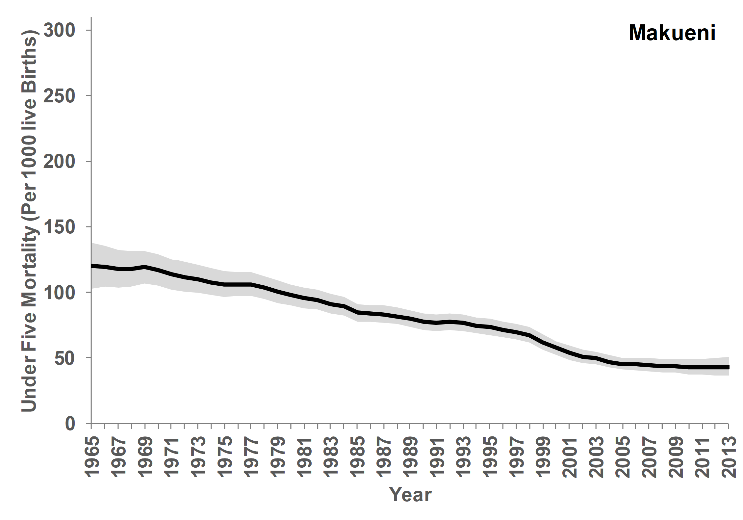


#### Central Province


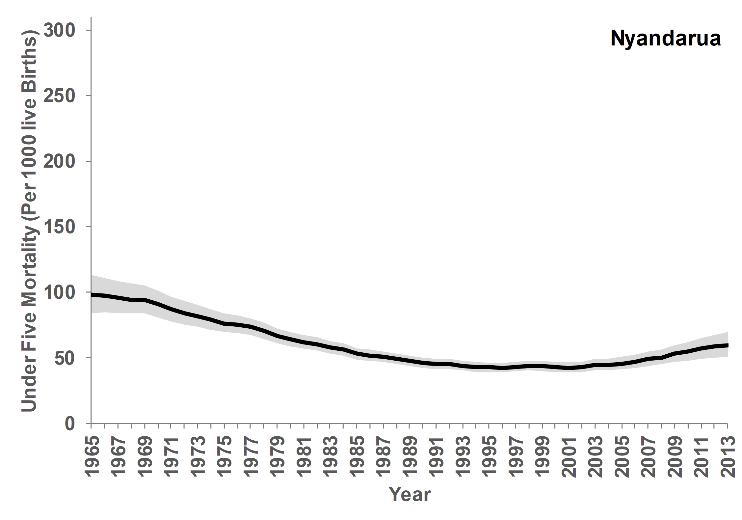

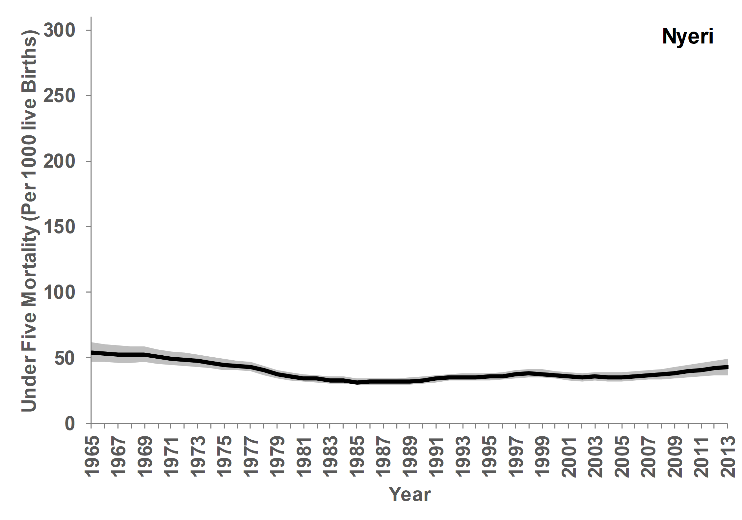


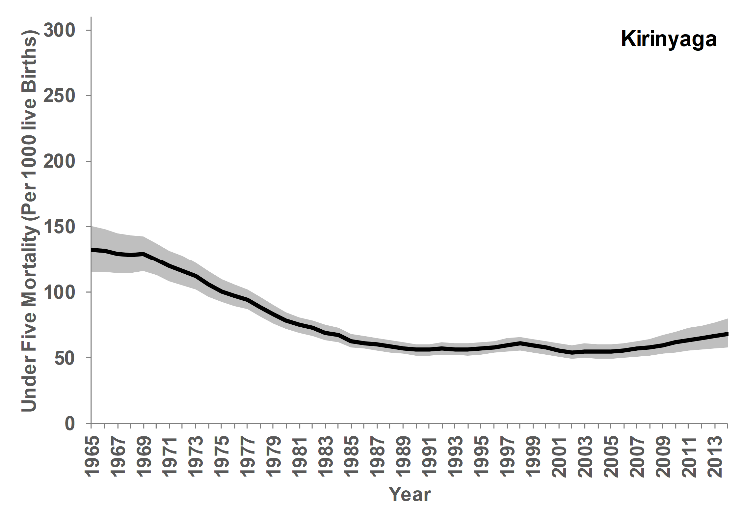

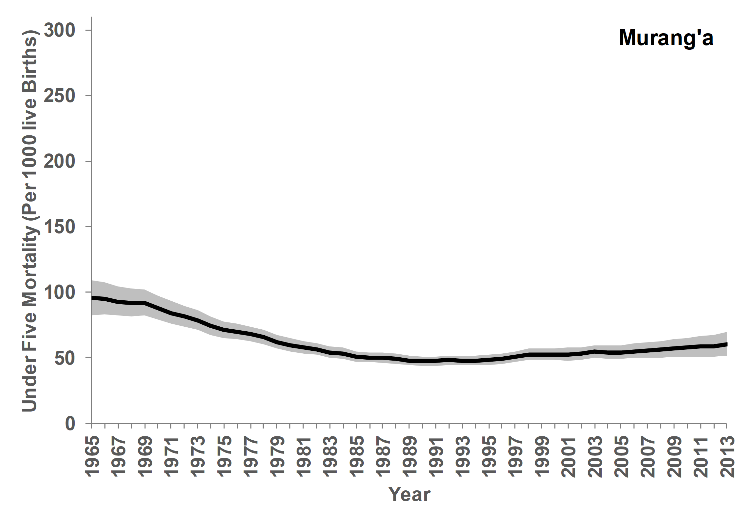


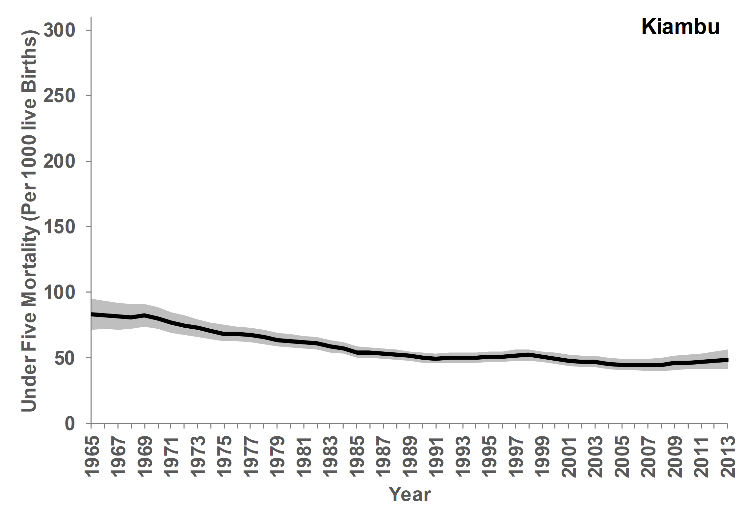


#### Rift Valley Province


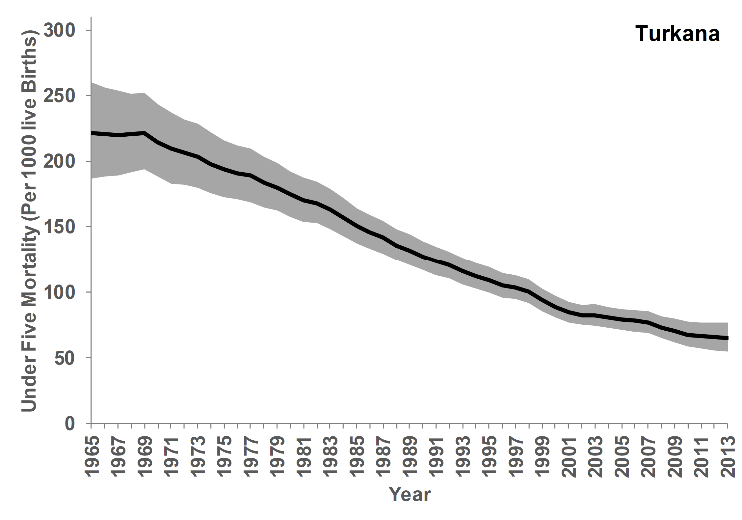

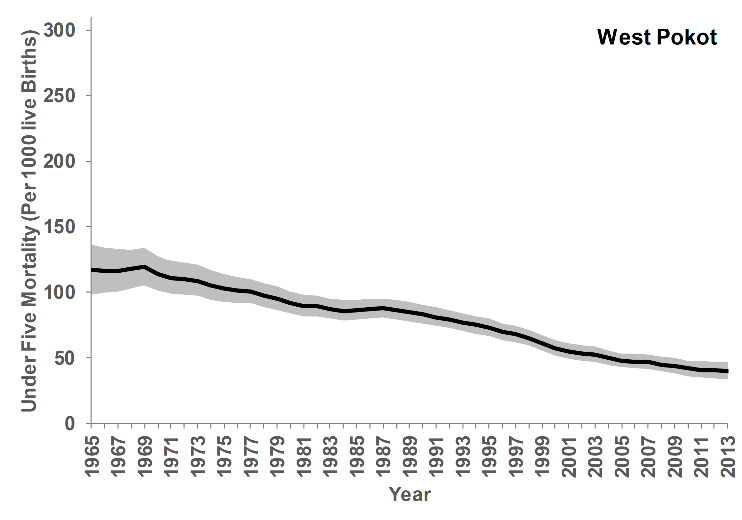


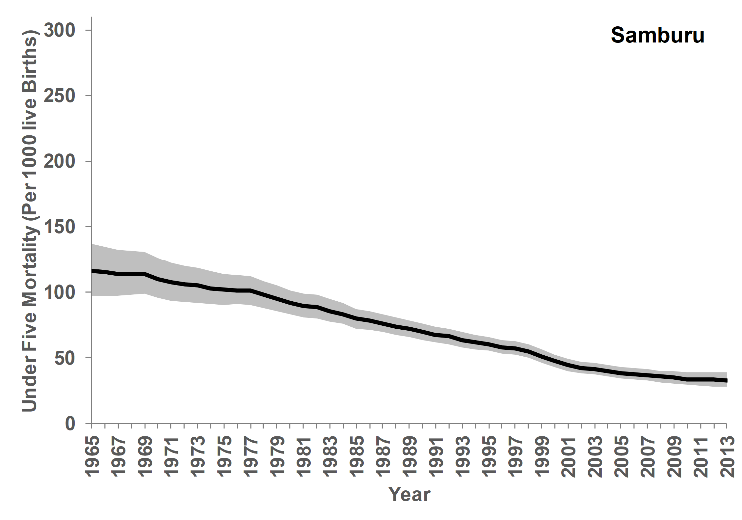

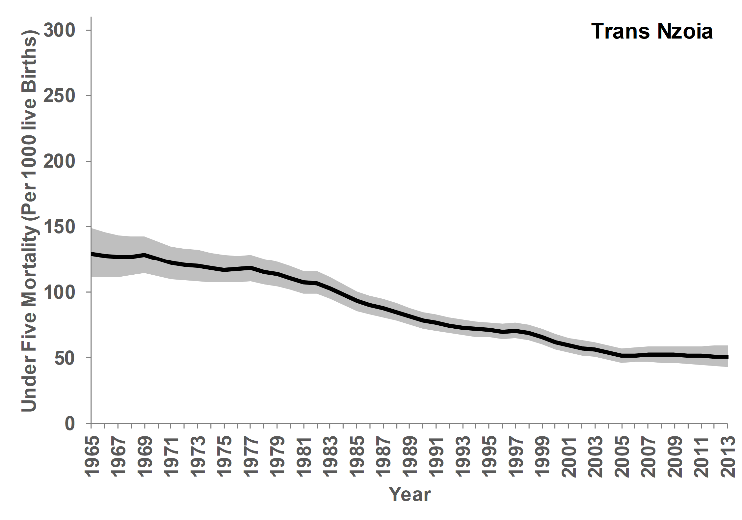


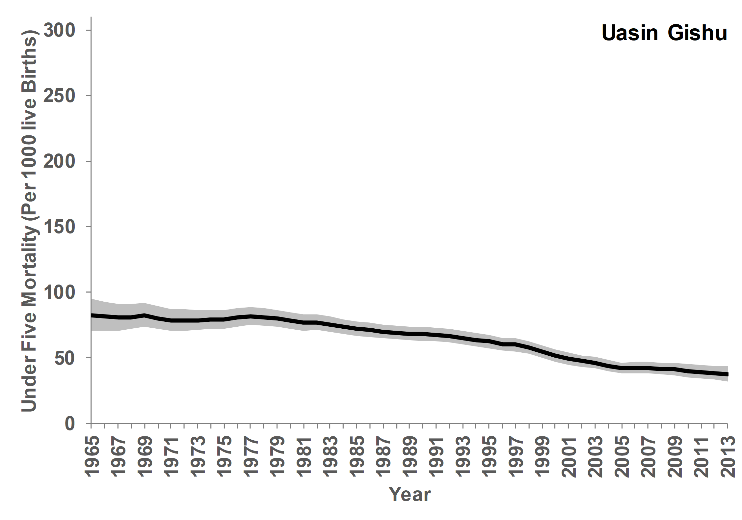

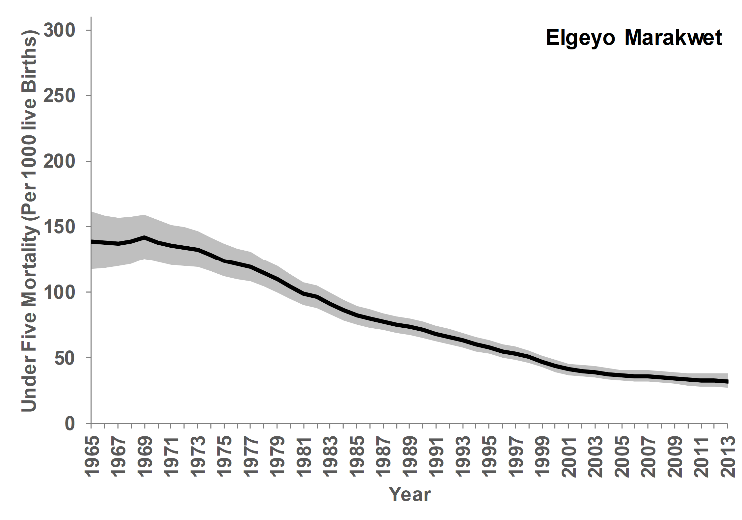


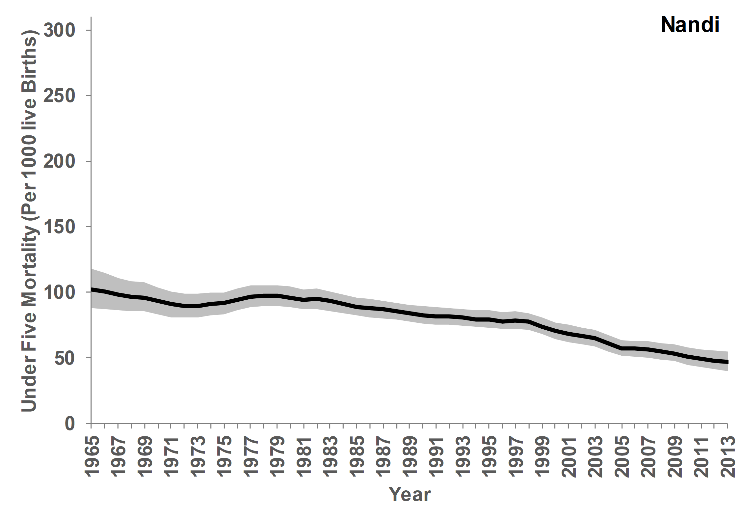

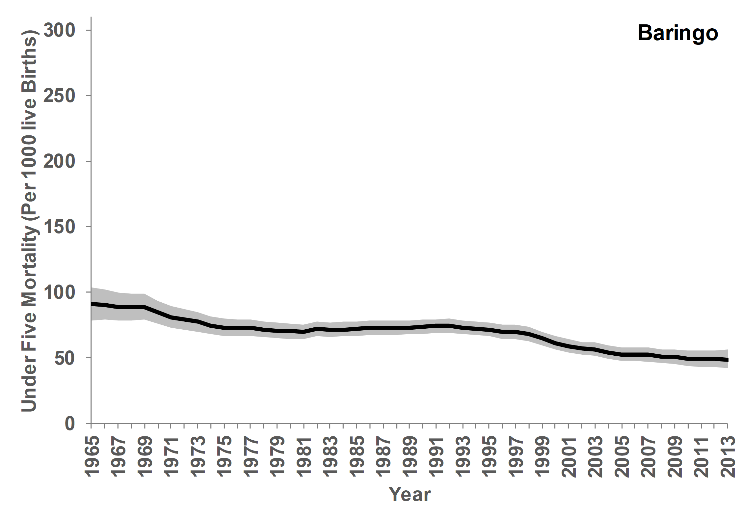


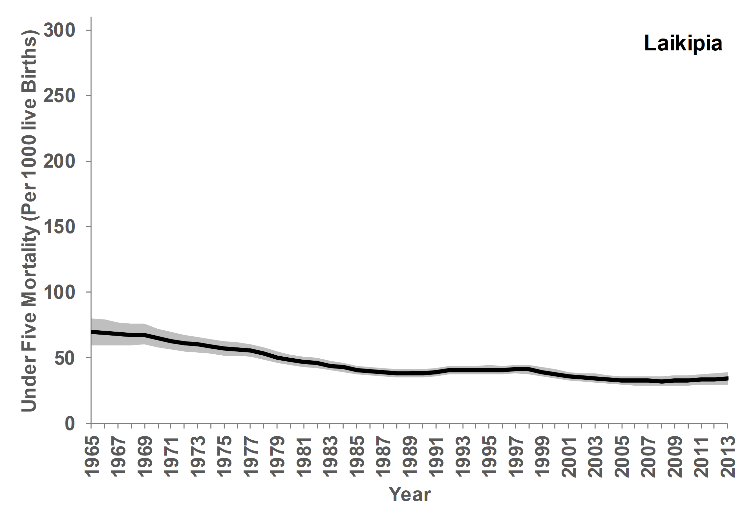

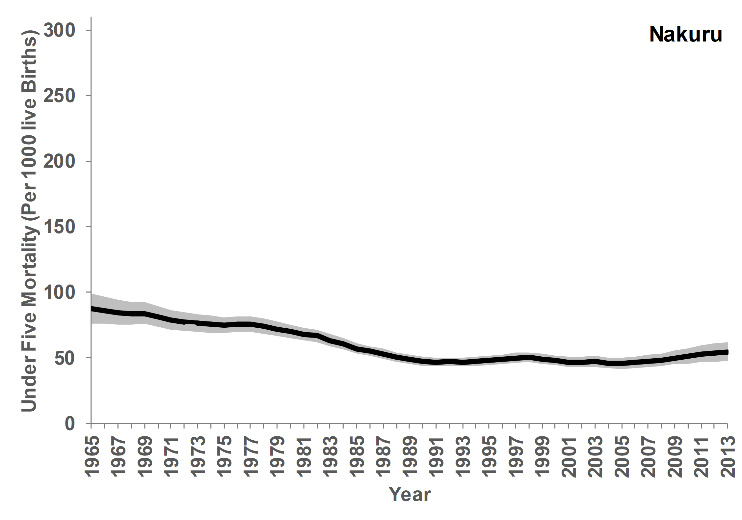


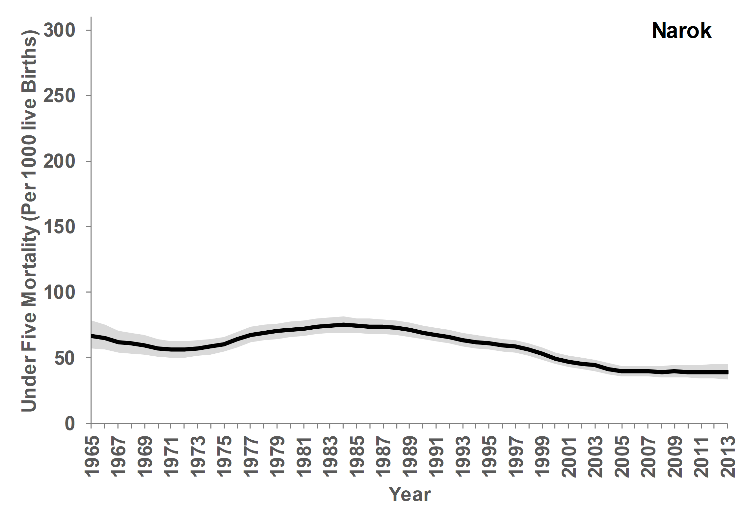

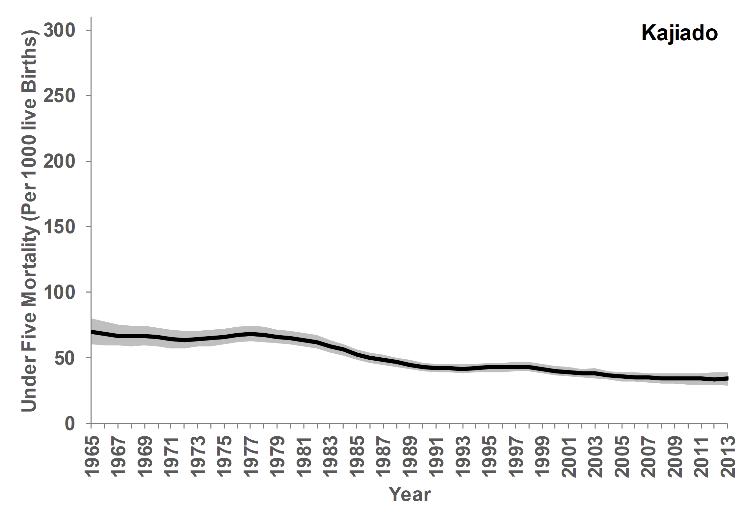


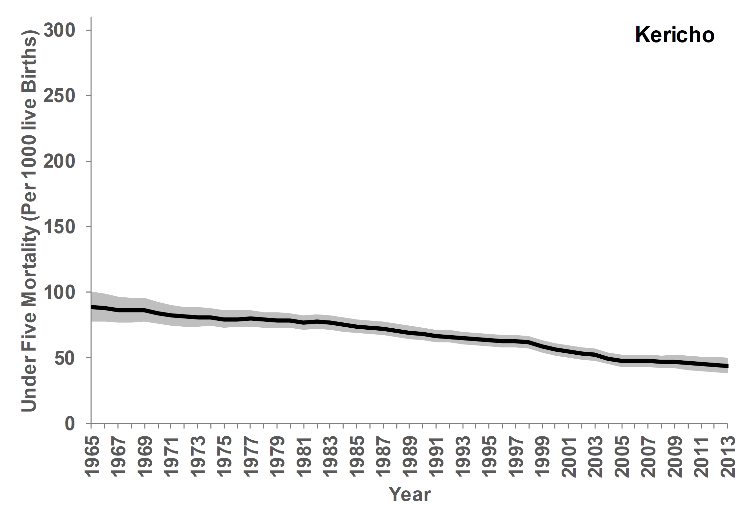

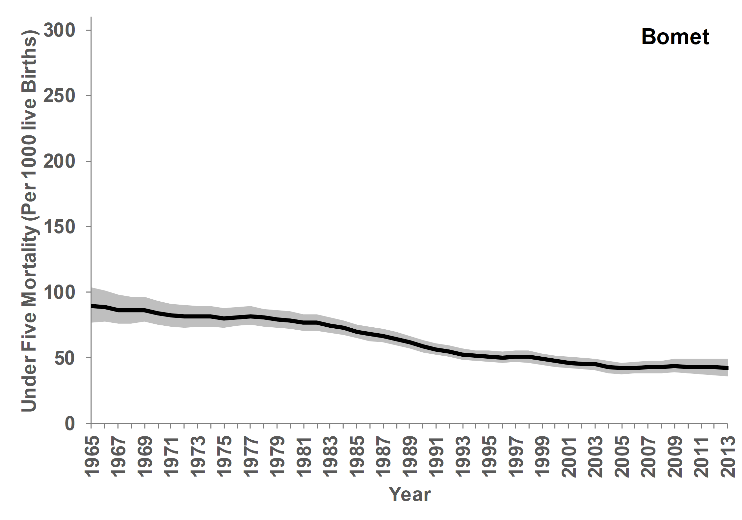


#### Western province


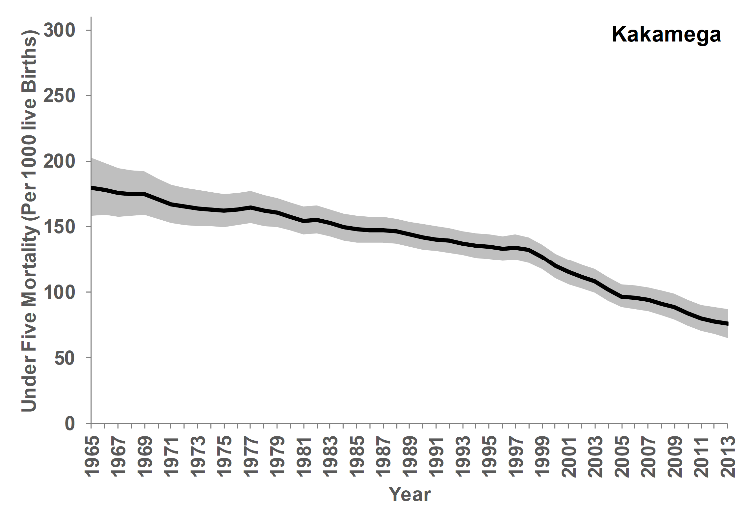

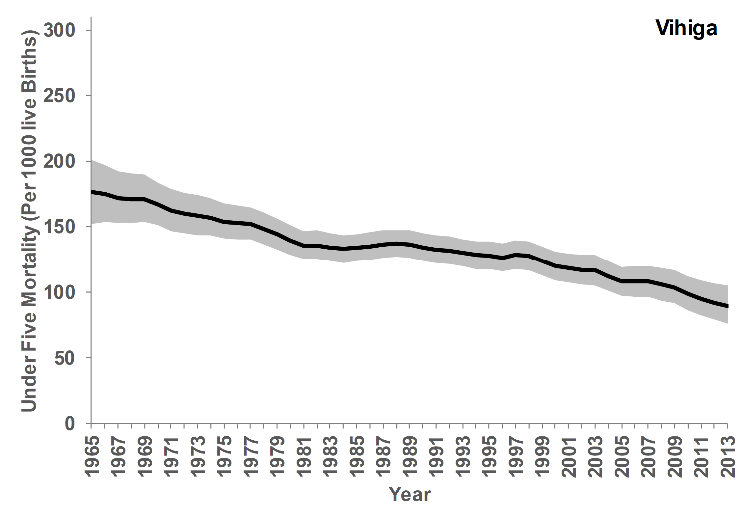


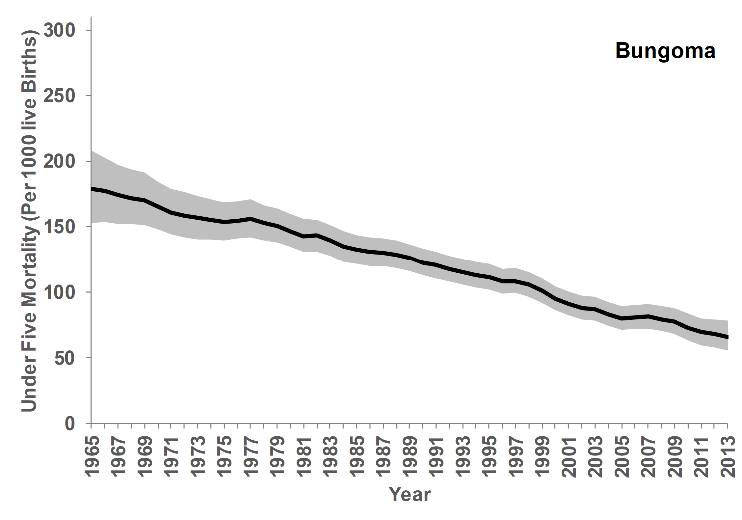

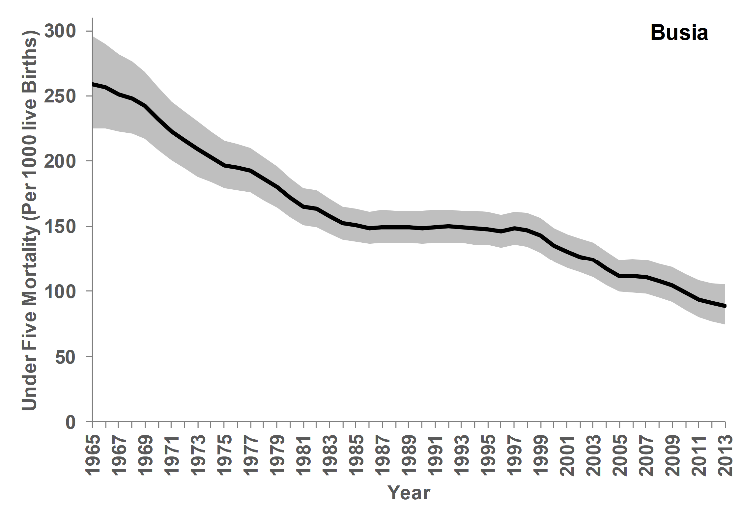


#### Nyanza Province


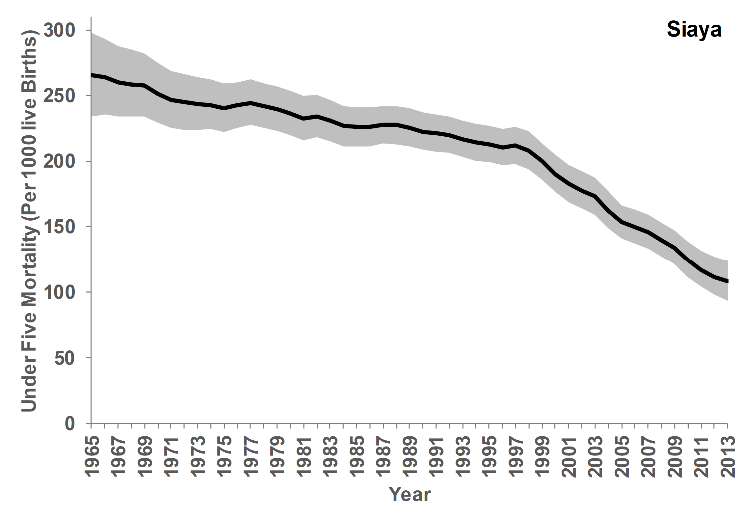

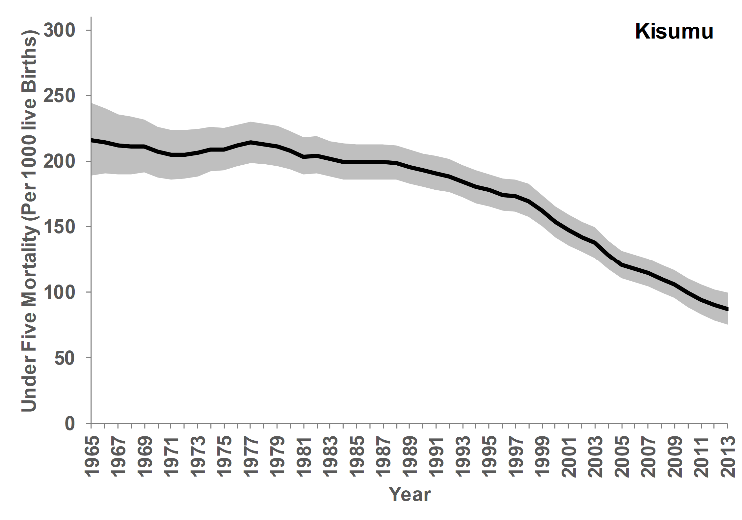


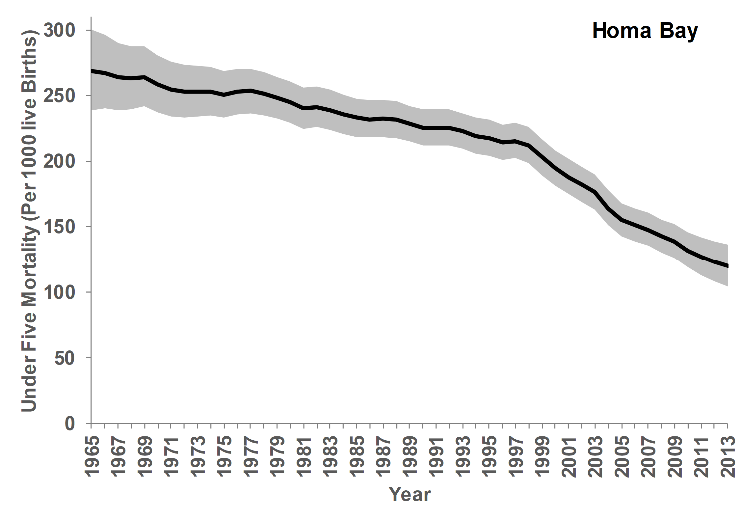

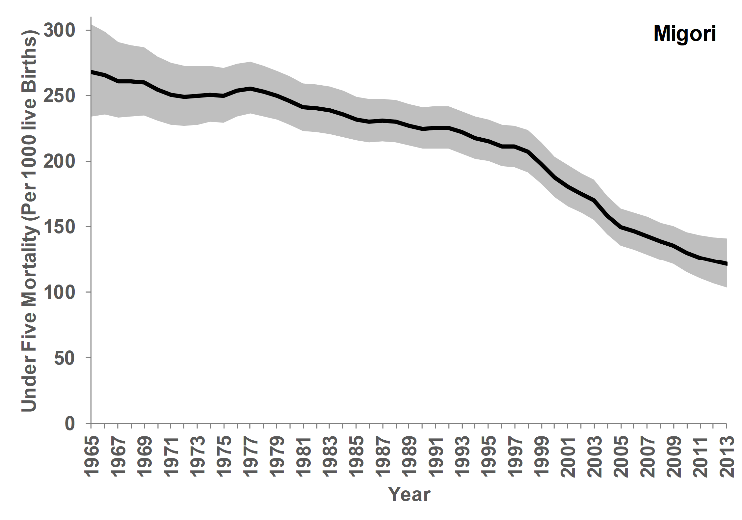


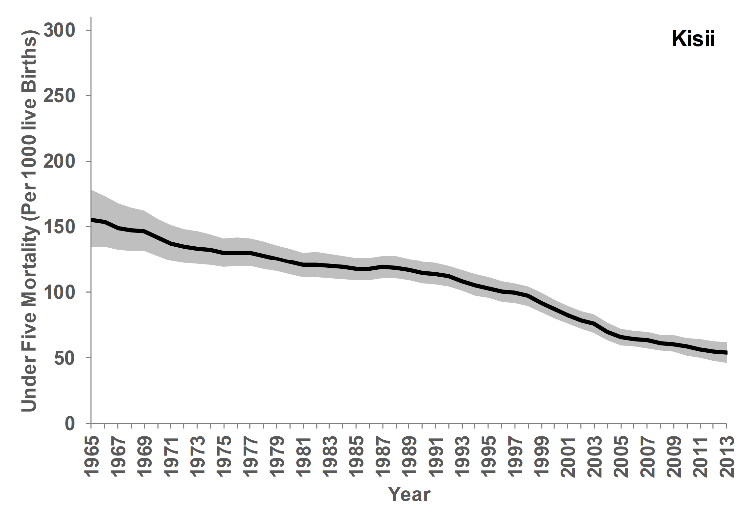

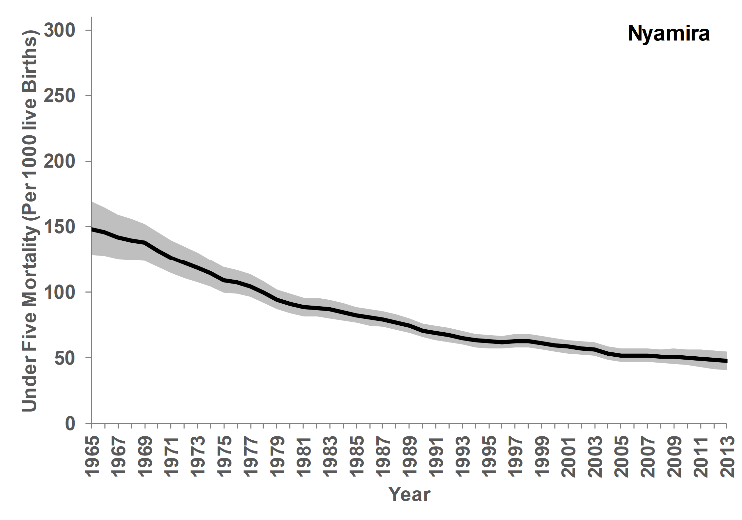


#### Nairobi Province/County


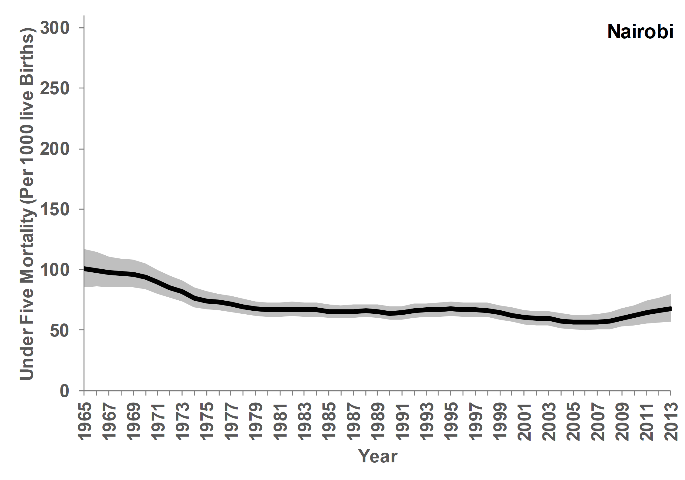

Supplement: Supplementary file 5 — Graphs of county level mean under five mortality and the corresponding 2·5–97·5% interquartile credibility range in Kenya. (DOCX 1378 kb) [file 12889_2019_6474_MOESM5_ESM.docx]
